# Optimization of a GC-MS method for the profiling of microbiota-dependent metabolites in blood samples: An application to type 2 diabetes and prediabetes

**DOI:** 10.3389/fmolb.2022.982672

**Published:** 2022-09-23

**Authors:** Patrycja Mojsak, Katarzyna Maliszewska, Paulina Klimaszewska, Katarzyna Miniewska, Joanna Godzien, Julia Sieminska, Adam Kretowski, Michal Ciborowski

**Affiliations:** ^1^ Clinical Research Centre, Medical University of Bialystok, Bialystok, Poland; ^2^ Department of Endocrinology, Diabetology and Internal Medicine, Medical University of Bialystok, Bialystok, Poland

**Keywords:** GC-MS, optimization, gut microbiota, T2DM, plasma, serum

## Abstract

Changes in serum or plasma metabolome may reflect gut microbiota dysbiosis, which is also known to occur in patients with prediabetes and type 2 diabetes (T2DM). Thus, developing a robust method for the analysis of microbiota-dependent metabolites (MDMs) is an important issue. Gas chromatography with mass spectrometry (GC–MS) is a powerful approach enabling detection of a wide range of MDMs in biofluid samples with good repeatability and reproducibility, but requires selection of a suitable solvents and conditions. For this reason, we conducted for the first time the study in which, we demonstrated an optimisation of samples preparation steps for the measurement of 75 MDMs in two matrices. Different solvents or mixtures of solvents for MDMs extraction, various concentrations and volumes of derivatizing reagents as well as temperature programs at methoxymation and silylation step, were tested. The stability, repeatability and reproducibility of the 75 MDMs measurement were assessed by determining the relative standard deviation (RSD). Finally, we used the developed method to analyse serum samples from 18 prediabetic (PreDiab group) and 24 T2DM patients (T2DM group) from our 1000PLUS cohort. The study groups were homogeneous and did not differ in age and body mass index. To select statistically significant metabolites, T2DM vs. PreDiab comparison was performed using multivariate statistics. Our experiment revealed changes in 18 MDMs belonging to different classes of compounds, and seven of them, based on the SVM classification model, were selected as a panel of potential biomarkers, able to distinguish between patients with T2DM and prediabetes.

## Introduction

The worldwide prevalence of type 2 diabetes mellitus (T2DM) has risen over the past two decades (Sobczak et al., 2019) and currently, this metabolic disease is a serious public health problem (Fikri et al., 2020). There is an increasing evidence that alterations in gut microbiota (GM) (Lin et al., 2017; Moon et al., 2018; Chen et al., 2019), apart from genetic (Mojsak et al., 2021) and life style factors (Ericson et al., 2018), are important for the development of metabolic diseases. Changes in the gut microbiome composition lead to an imbalanced gastrointestinal habitat which promotes abnormal production of metabolites, inflammatory status, glucose metabolism alteration and even insulin resistance (IR) (Tanase et al., 2020). Particularly, various microbiota-dependent metabolites (MDMs) (Han et al., 2021), such as short–chain fatty acids, branched–chain fatty acids, amino acids (AAs), branched–chain amino acids (BCAAs), bile acids, tryptophan-derived metabolites, and others (Mojsak et al., 2022; Zhou et al., 2022) have been reported to be closely associated with IR (Menni et al., 2022), prediabetes (Dorcely et al., 2017; Gar et al., 2018) and T2DM (Sobczak et al., 2019; Fikri et al., 2020; Gu et al., 2020; Mojsak et al., 2021).

Metabolomics is a high–throughput approach enabling a global analysis of metabolites in biological systems. Untargeted metabolomics has led to many discoveries of microbiota-dependent metabolic pathways and metabolites linked to host diseases (Moon et al., 2018; Tanase et al., 2020; Mojsak et al., 2022). Consequently, determination of MDMs can be essential for the early diagnosis of T2DM (Mojsak et al., 2022). Among mass spectrometry (MS)–based analytical platforms, gas chromatography (GC–MS) and liquid chromatography (LC-MS) are the most popular analytical techniques used for the separation of MDMs (Chen et al., 2019; Eylem et al., 2022). Compared to LC, GC has a considerably better chromatographic resolution. It is also a highly reproducible and sensitive analytical technique, able to detect a wide variety of MDMs such as AAs, fatty acids (FAs), carbohydrates (CARBs) and sterols related to microbiota and T2DM, that would otherwise need several separations in LC-MS (Heaney, 2020). Additionally, reproducible molecular fragmentation patterns of GC-MS make it one of the most reliable tools for exploring metabolites (Papadimitropoulos et al., 2018). Considering all above-mentioned reasons, GC-MS was used in this study.

MDMs are found in a variety of biological samples such as feces, urine, serum or plasma (Chen et al., 2019). According to Chen et al. (2019), plasma and serum are non–invasively obtained biomatrices and analysis of their metabolome may reflect the changes in the metabolome of the whole organism (Kiseleva et al., 2021), including changes in MDMs composition (Vernocchi et al., 2016). Additionally, both of these blood-derived samples have been used in many GC-MS metabolomics studies related to the development of T2DM (Lin et al., 2017; Chen et al., 2019). Visconti et al. (2019), proved that many species of GM showed association with blood metabolites, suggesting important effects on host systemic metabolism.

Although GC-MS is a powerful tool to detect MDMs, GC-MS profiling of such metabolites in biofluid samples constitutes an analytical challenge (Mojsak et al., 2022). In order to extend the coverage of MDMs measured with GC-MS, selection of a suitable solvent for the protein precipitation or extraction (He et al., 2021) and a transformation of analysed metabolites into more volatile forms through the derivatisation process, is required (Moldoveanu and David, 2018). Many solvents like ACN (Mojsak et al., 2021), MeOH (Trygg et al., 2005) or mixtures of solvents such as: ACN:isoProp:H_2_O (v:v:v; 3:3:2) (Fiehn, 2016; Kiseleva et al., 2021); MeOH:H_2_O (v:v; 9:1) (Malm et al., 2016), MeOH:isoProp:H_2_O (v:v:v; 3:3:2) (Kiseleva et al., 2021) or MeOH:EtOH (v:v; 1:1) (Szeremeta et al., 2021) are commonly used in metabolomics studies. A choice of proper solvent(s) is important, in the study of Fiehn (2016) it was confirmed that the use of hydrophilic, lipohilic and medium–polarity solvents demonstrated high analytical precision and comprehensiveness of the extracted metabolome. There are a few studies comparing the utility of different solvents, however, the variety of investigated mixtures was not that wide (Trygg et al., 2005; He et al., 2020; Eylem et al., 2022). To the best of our knowledge, there is a lack of methodological articles comparing the performance of these solvents and their mixtures for the preparation of serum or plasma samples for the GC-MS analysis of 75 MDMs.

Out of several available derivatisation methods (Moldoveanu and David, 2018), methoxymation (MeOx) followed by silylation (SIL) is the most commonly used (Fritsche-Guenther et al., 2021). According to Fiehn (2016) and Eylem et al. (2022), prior to SIL, MeOx is necessary to protect carbonyl groups of aldehydes and ketones in reducing sugars from the cyclization process, as well as to stabilize α–keto acids against decarboxylation (Zarate et al., 2016). The degree of completion of this process is associated with the concentration and volume of methoxamine HCl in pyridine, as well as incubation time and temperature used during the derivatisation process (Bekele et al., 2014; Moros et al., 2017). The most frequently reported incubation time and temperature for MeOx of biofluid metabolites are 30–90 min at 37–70°C or 16 h at room temperature (RT) (Fiehn, 2016; Beale et al., 2018), whereas the volumes of methoxamine HCl in pyridine used are between 10 and 40 µl (with the concentration range of 15–40 mg/ml) (Miyagawa and Bamba, 2019). As for the MeOX process, the main factors determining the outcomes of the GC–MS analysis are the volume of SIL reagent as well as the incubation time and temperature (Eylem et al., 2022).

To the best of our knowledge no thoroughly optimized method, which includes the impact of popular solvents and derivatisation conditions for profiling of 75 MDMs present in human plasma and serum has been presented so far. For this reason, we performed a side–by–side comparison of different serum and plasma samples preparation procedures composed of solvent-based simultaneous protein precipitations with metabolites extraction and two-step derivatisation, MeOx followed by SIL, focusing on MDMs. Based on the signal intensity and its relative standard deviation (RSD), the methodology for sample preparation and analysis was optimized to obtain the best possible method for the analysis of MDMs. Metabolites were classified as MDMs based on the scientific reports (Lin et al., 2017; Org et al., 2017; Martin et al., 2019; Visconti et al., 2019; Wan et al., 2020; Gojda and Cahova, 2021; Tan et al., 2021) and Human Metabolome Database (HMDB) (HMDB, 2020) (http://www.hmdb.ca accessed, on 20 April 2022). Finally, we used the developed biofluids preparation method to analyse serum samples of patients with prediabetes and T2DM.

## Material and methods

### Reagents

The following reagents and standards were used: MilliQ^®^ water (Millipore, Billerica, MA, United States), heptane (Sigma–Aldrich, Steinheim, Germany), pyridine (Sigma–Aldrich, Steinheim, Germany), O–methoxyamine HCl (Sigma–Aldrich, Steinheim, Germany) and MSTFA (N-Methyl-N-trimethylsilyl-trifluoroacetamide) with 1% TMCS (Trimethylchlorosilane) (Pierce Chemical Co., Rockford, IL, United States). 4–nitrobenzoic acid (4–NBA) and stearic acid methyl ester (C18:0 methyl ester) (Sigma–Aldrich, Steinheim, Germany) were used as internal standards (ISs) for GC–MS. Individual stock solutions of 4–NBA (IS1) were prepared at the concentration of 25 ppm, then stored at −4°C, whereas methyl stearate (IS2) were prepared at the concentration of 20 ppm, then stored at −20°C. Two standards mixtures for GC–MS, one containing grain fatty acid methyl esters (FAME) (C8:0—C22:1, n9) and another standard mix with mixture of n–alkanes (C8:C40) were purchased from Supelco (Bellefonte, PA, United States).

### Optimization of sample preparation for GC–MS analysis

At the optimization stage, plasma and serum samples, as well as all tested combination of parameters, were analysed in separate sequences. Three replicates were used for each optimized condition. In the batches tested samples were analysed together with quality control samples (QCs) and blank samples. Firstly, 50 μl of pooled human plasma or serum were extracted with 150 µl of ACN, ACN:isoProp:H_2_O (v:v:v; 3:3:2), MeOH:H_2_0 (v:v; 9:1) MeOH:isoProp:H_2_O (v:v:v; 3:3:2) and MeOH:EtOH (v:v; 1:1) in order to evaluate the performance of different extraction solvents. Subsequently, a two–step derivatisation: 1) MeOX with 20 µl O–methoxyamine HCl in pyridine (15 mg/ml, for 30 min at 37°C) followed by 2) SIL with 20 µl MSTFA containing 1% TMCS (for 30 min at 37°C) was performed.

To optimize the protocol for two-step derivatisation process, firstly, we tested different concentrations (10–40 mg/ml, in the volume of 10 μl) and then volumes (10–50 µl) of methoxyamine HCl in pyridine, adjusted to 120 μl with heptane containing IS2. After that, we examined the effects of derivatisation temperature and time of the reaction. For MeOx, the derivatisation temperature was set to 37°C or 70°C, and the derivatisation time was set to 30 min, 60 min or 16 h at RT [30 min at 37°C (P1), 1 h at 70°C (P2), 16 h at RT (P3) and 1 h at 70°C followed by 16 h at RT (P4)]. In the first part of the experiment, SIL conditions were fixed at 30 min and 37°C. Then, when SIL step was being optimized, the derivatisation temperature of 37 and 70°C (for 30 and 60 min each) was evaluated. Finally, the optimized method was applied to the analysis of the clinical samples.

### Clinical samples preparation

Serum (50 µl) was deproteinised with 150 µl (MeOH:H_2_0, 9:1, v:v) (1:3, −20°C) containing IS1, followed by two–step derivatisation: 1) MeOX with O—methoxyamine HCl in pyridine (30 mg/ml, RT, 16 h) followed by 2) SIL with MSTFA containing 1% TMCS (70°C, 1 h). Subsequently, sample preparation for QC samples was performed as described above for clinical samples. Preparation of a blank was conducted following the same procedure, but using only solvents. Six QC samples were injected to equilibrate the analytical platform before clinical samples were analysed to ensure that reproducible data was acquired. In each batch, FAMEs, mixes of n-alkanes, blank and six QC samples were injected at the beginning of the batch, and one QC sample were injected after every eight sample injections. At the end of the batch, one QC sample and blank were injected again.

Metabolic fingerprinting was performed using GC system (series 7890B) equipped with an 7693A autosampler and a Mass Selective Detector 7000D (Agilent Technologies, Palo Alto, CA, United States). One µl of the derivatised serum sample with ISs was injected into a DB–5MS capillary GC column (30 m × 0.25 mm × 0.25 µm) using helium as a carrier gas at a constant gas flow of 1.0 ml/min. The injector temperature was set to 250°C and the split ratio to 1:10. The temperature gradient program started at 60°C, was held for 1 min, followed by a subsequent increase in temperature to 320°C at a rate of 10°C/min. The GC–MS transfer line, filament source and the quadrupole temperature were set to 280, 230, and 150°C, respectively. The electron ionisation source was set to 70 eV, and the mass spectrometer was operated in the full scan mode, applying a mass range from m/z 50 to 600 at a scan rate of 1.38 scan/s.

### Untargeted GC–MS data analysis

The deconvolution and identification were performed using Mass Hunter Quantitative Unknowns Analysis software (B.07.00, Agilent), alignment with Mass Profiler Professional software (version 13.0, Agilent) and peak integration using Mass Hunter Quantitative Analysis software (version B.07.00, Agilent). The identification was performed mainly based on the accurate mass and product ion spectrum matching against in–house library of 100 authentic standards as well as Fiehn’s and NIST 14 libraries. Prior to the statistical analysis, clinical sample areas were normalised by IS abundance in order to minimise the response variability coming from the instrument. Finally, data were filtered based on the coefficient of signal variation (CV) in QC samples, considering values lower than 30% as acceptable.

In order to perform the differential analysis of the metabolomics data, the variables were then filtered as proposed by Godzien et al. (2015). Missing values were replaced by k–means nearest neighbour (Armitage et al., 2015) using the in–house built scripts for MATLAB 7.10 R2010a (MathWorks Inc., Natick, MA, United States)).

### Sample collection

For the first step of optimization, plasma and serum samples were collected from the same individuals. For plasma samples, blood was collected to S-Monovette K3EDTA tubes (SARSTEDT, Germany) and plasma was obtained after centrifugation at 15,400 x g for 10 min at 4°C. For serum samples, blood was collected to S-Monovette tubes containing clot activator and tubes were stored in the vertical position at RT for 60 min to allow the formation of a clot. Afterwards, tubes were centrifuged in a horizontal rotor (swing–out head) for 10 min at 1,300 x g at RT. After centrifugation, serum or plasma fraction was transferred to Eppendorf tubes and stored at −80°C until the day of analysis. All the procedures were approved by the Local Ethics Committee of the Medical University of Bialystok (Permit No. R–I–002/193/2019). All donors signed informed consent.

For this experiment, 24 individuals with T2DM and 18 with prediabetes were selected from the 1000PLUS cohort, gathered between 2014 and 2017 by the Department of Endocrinology, Diabetology and Internal Medicine, Medical University of Bialystok, Poland (Maliszewska et al., 2019). Ethical approval for the study was obtained from the local Ethics Committee at the Medical University of Bialystok, Poland (R–I–002/290/2008/2009, R–I–002/35/2014, and APK.002.239.2022). The presence of T2DM based on the dysglycemia diagnostic criteria of the Diabetes Poland was confirmed or excluded using glucose concentration measurements during an oral glucose tolerance test (OGTT) at 0 and/or 120 min (Diabetology, 2018). Table 1 presents the median and the range of the anthropometric measurements and biochemical parameters. Presented *p*-value was calculated using the Mann–Whitney *U*-test.

**TABLE 1:** Characteristics of the studied group (median and range).

Clinical parameters	PreDiab *N* = 18	T2DM *N* = 24	P–value
Age [years]	56.39 (37.36–70.96)	62.5 (41.16–69.20)	0.146
Female/Male	**8/10**	**11/13**	
BMI [kg/m2]	33.55 (23.66–47.05)	32.51 (21.25–49.35)	0.219
Fasting glucose 0 min [mg/dL]	110 (101–121)	131 (138–171)	0.0018
Glucose 120 min [mg/dL]	126 (72–190)	206 (160–229)	0.0001
Insulin [µU/mL]	126 (72–190)	16.35 (4.73–58.81)	0.880
HbA1c [%]	5.8 (5.10–6.40)	6.15 (5.3–7.7)	0.0057
LDL cholesterol [mg/dL]	105.1 (53.6–221.6)	93.8 (60.4–213.40)	0.348
Total cholesterol [mg/dL]	181 (125–284)	173.5 (138–310)	0.723
HDL cholesterol [mg/dL]	49.70 (29–125)	52 (36–88)	0.319
Triglyceride [mg/dL]	107 (33–229)	124.5 (44–232)	0.875
HOMA–IR	4.30 (2.80–10.40)	5.20 (1.10–20.00)	0.479
HOMA–B	112.00 (71.00–216.00)	85.00 (19.00–277.00)	0.112

BMI, body mass index; HbA1c, glycated hemoglobin A1c; LDL cholesterol, high-density lipoprotein cholesterol; HDL cholesterol, high-density lipoprotein cholesterol; HOMA-IR, homeostasis model assessment for insulin resistance; HOMA-B, homeostasis model assessment for beta (*β*) cell function, p-value—difference between control and T2DM (based on the Mann–Whitney *U* test). PreDiab-subjects with prediabetes, T2DM-subjects with T2DM.

### Repeatability, reproducibility and stability of 75 MDM measurements

In order to identify the optimal conditions the following criteria were taken into account 1) repeatability, 2) the peak intensities of individual metabolites 3) the total intensity (TI) of all metabolites. Finally, reproducibility and stability were determined. We used the relative standard deviation (RSD) (defined as the [(standard deviation)/(mean) × 100]) of metabolite abundance for evaluation of the consistency of metabolites measurement using a GC–MS platform. A commonly accepted maximum tolerance of RSD for GC–MS in metabolomics studies is 30% (Koek et al., 2011). The stability, repeatability and reproducibility of the analysis of 75 MDMs measurement were assessed by calculating RSD of the GC-peak area. To test the repeatability, 50 replicates (*n* = 50) of human plasma and serum were analysed using optimal parameters. To test the reproducibility, 10 quality control samples (*n* = 10) (plasma or serum samples) were analysed over three batches (s = 3) within the scope of a biological study, i.e., realistic conditions (Supplementary Table S2). To test the stability, the plasma or serum samples were re–injected after 8, 24, 36, and 48 h (Supplementary Table S3).

### Statistical analysis

To select statistically significant metabolites between the PreDiab and T2DM groups multivariate statistics using an orthogonal projections to latent structures discriminant analysis (OPLS–DA) was used. Statistically significant metabolites were chosen based on the predictive loading value [p (corr)] and variable importance in the projection (VIP) value. Validation of the OPLS–DA models was performed by cross–validation using the leave 1/3 out approach as described previously (Ciborowski et al., 2012). Multivariate statistics was performed in SIMCA−P + 13.0.3.0 (Umetrics, Umea, Sweden). For statistically significant metabolites, the receiver operating characteristic (ROC) analysis was conducted in MetaboAnalyst 5.0.

## Results

### Optimization step

Initially, based on the literature review (Lin et al., 2017; Org et al., 2017; Martin et al., 2019; Visconti et al., 2019; Wan et al., 2020; Gojda and Cahova, 2021; Tan et al., 2021) and information provided in HMDB (www.hmdb.ce, access on 20^th^ April 2022) we prepared a database of MDMs and metabolites involved in pathways impacted by GM (Supplementary Table S1). Then, we performed GC-MS analyses of pooled plasma or serum samples to check which MDMs are present in these two matrices. As a result, we observed 335 and 348 raw peaks in plasma and serum, respectively. After data pretreatment (deconvolution, alignment, data normalization and filtering), 102 entities were obtained for both matrices, 85 metabolites could be identified taking into account several derivatives from one metabolite for some AAs and CARBs. Finally, we chose 75 MDMs, representing different analytical classes, with RSD below 30% in both plasma and serum samples (Supplementary Table S1).

### Selection of extraction solvent

**FIGURE 1:**
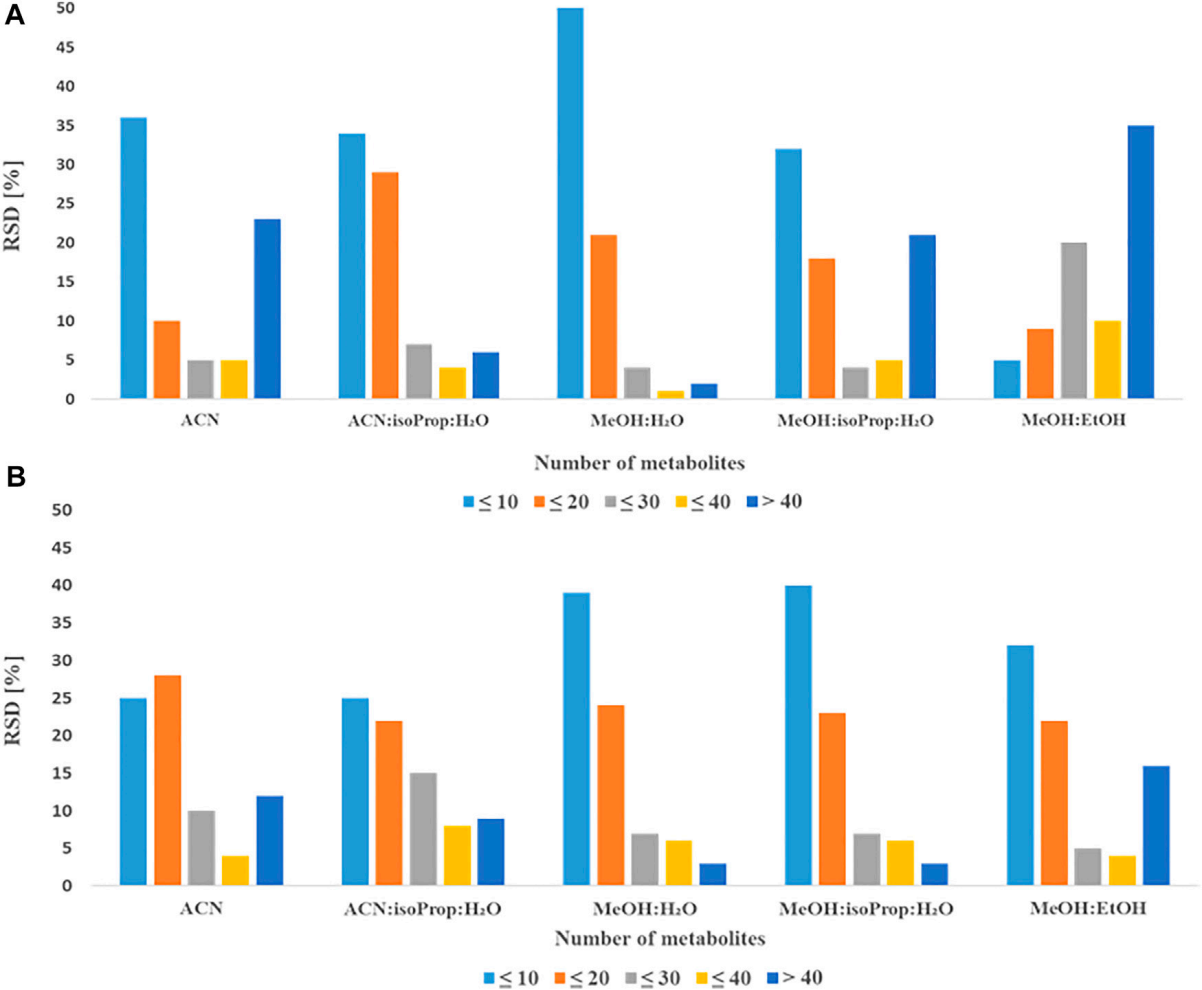
Comparison of obtained RSDs for all detected metabolites extracted with different solvents from plasma **(A)** or serum **(B)**.

**FIGURE 2:**
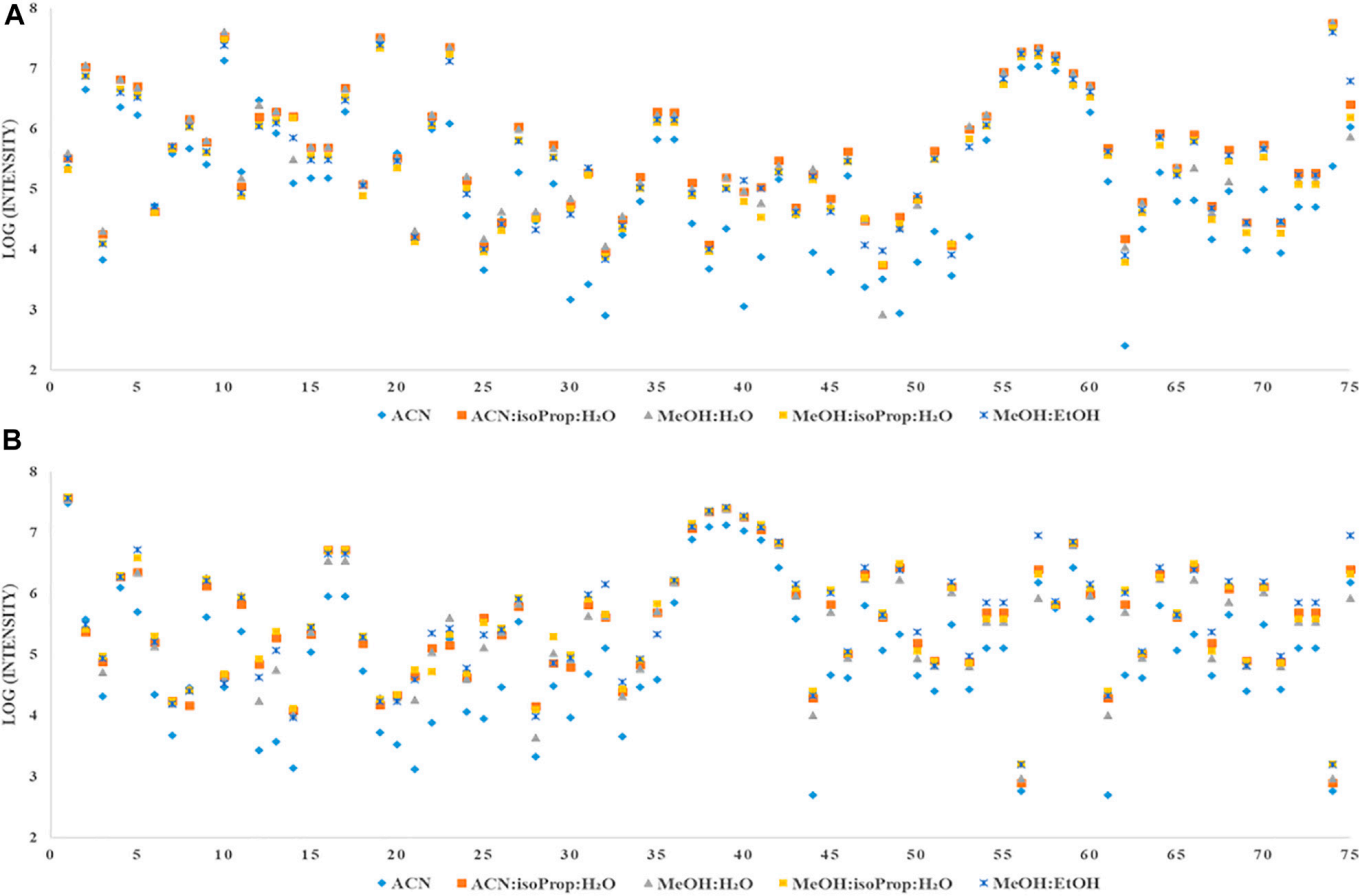
Comparison of intensity of all detected metabolites extracted with different solvents from plasma **(A)** or serum **(B)**.

To select the best solvent for protein precipitation and extraction of plasma and serum samples, four widely used mixtures of solvents: AcN:isoProp:H2O (v:v:v; 3:3:2) MeOH:H2O (v:v; 9:1); MeOH:isoProp:H_2_O (v:v:v; 3:3:2), MeOH:EtOH (v:v; 1:1) (hereinafter referred to as solvents) and ACN alone were tested. In the Figures 1, 2 we present repeatability and intensity (A-plasma, B-serum) obtained for MDMs measurement after the extraction with five combinations of solvents in both matrices. MeOH:H_2_O mixture (v:v; 9:1) and MeOH:isoProp:H_2_O mixture (v:v:v; 3:3:2) were selected as the best extraction solvents for both types of samples. The intensity of the signals for these two solvents were comparable but the extraction using MeOH:H_2_O mixture (v:v; 9:1) had better repeatability, and for this reason this solvent was chosen for the next step of optimization. In plasma samples extracted with MeOH:H_2_O mixture (v:v; 9:1), 21 MDMs had RSD below 20% and 51 below 10%. However, the RSD of 40 and 23 of all detected MDMs in serum were in the range of 0.5%–8.5% and 10.1%–19.7%, respectively. Additionally, the metabolite signals from ACN, MeOH:isoProp:H_2_O (v:v:v; 3:3:2) and MeOH:EtOH (v:v, 1:1) in both matrices had a wider range of RSD and a larger number of MDMs in the RSD range of 30%–40% and above 40% than those obtained with other extraction solvents.

**FIGURE 3:**
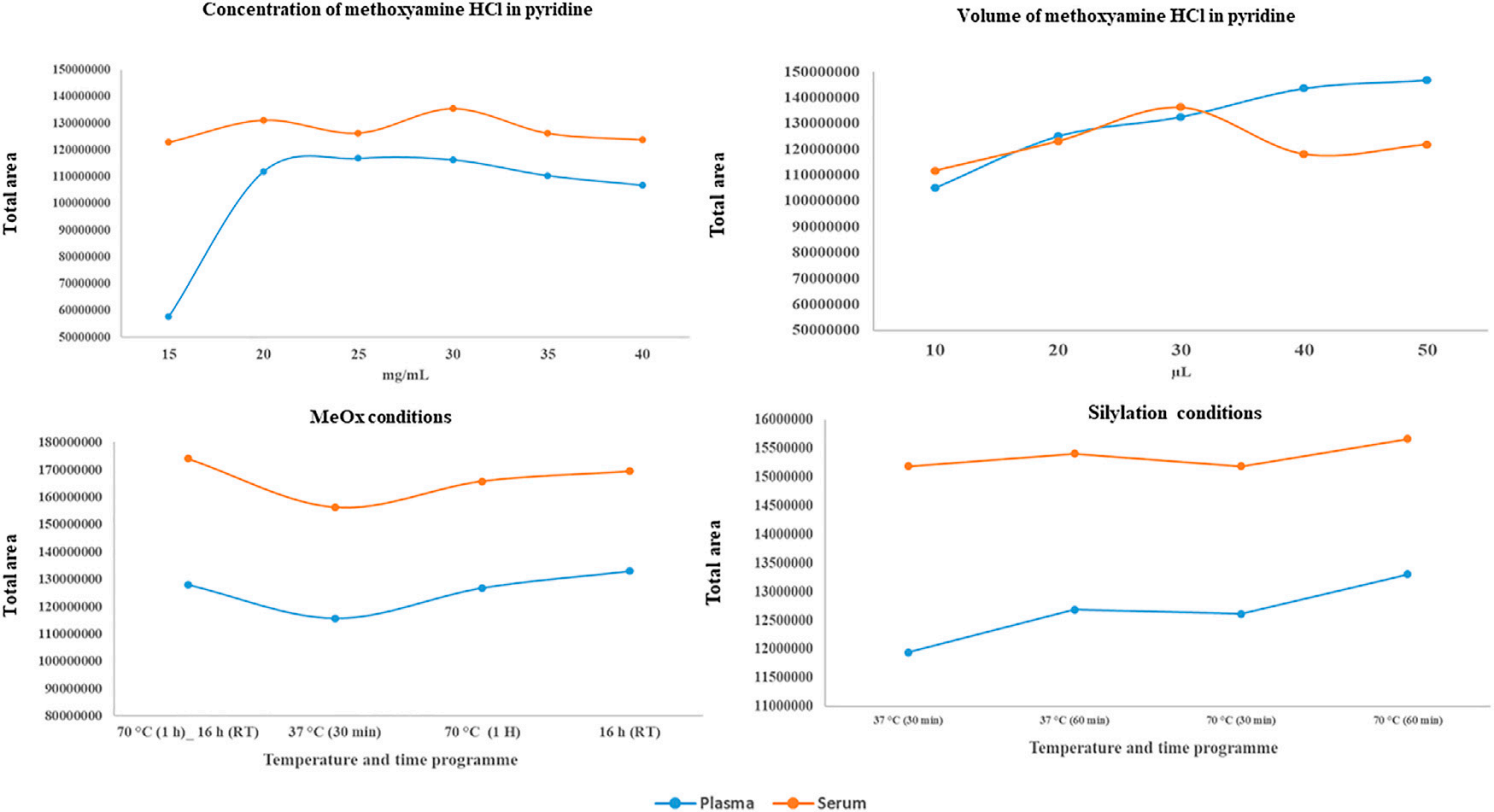
Effect of tested conditions on plasma and serum samples for all tested metabolites on the TI.

In the next step, we compared the impact of different concentrations and volumes of MeOx on the derivatisation process (see Figure 3). For MeOx, a solution of O–methoxyamine HCl in pyridine was employed. First, we compared six commonly used concentrations of O–methoxyamine HCl in pyridine (15, 20, 25, 30, 35, and 40 mg/ml, in the volume of 10 μl for both matrices, adjusted to 120 µl with heptane containing IS2). In plasma samples, the intensity of the majority of detected metabolites (46 from 75) increased with the elevated concentration of O-methoxyamine HCl in pyridine up to 30 mg/ml, with some exception for AAs (alanine (Ala), isoleucine (isoLeu), serine, methionine (Met), cysteine (Cys), aspargine, glutamine (Glu) and tyrosine). The intensity of this metabolites decreased when the concentration of O-methoxyamine HCl was higher than 30 mg/ml. In serum samples we observed the highest intensity at the MeOx reagent concentration of 30 mg/ml, higher concentrations resulted in similar intensity of metabolites with exception for creatinine (Cre) and AAs (Ala, phenylalalnine, Met and threonine (Thre). The intensity of these AAs decreased when the concentration of MeOx increased. TI of tested MDMs was the highest at the concentration of 30 mg/ml in serum samples. In plasma samples, the concentration of 25 mg/ml yields slightly higher TI of MDMs than 30 mg/ml but for the optimization, we have chosen universal concentration of 30 mg/ml of O-methoxyamine HCl in pyridine for two tested matrices. For chosen MeOX concentration the lowest median RSDs of 9.2% and 6.4% (Table 2) was indicated for plasma and serum, respectively. For the selected concentrations, RSD below 20% was found for 58 plasma and 71 serum metabolites.

**TABLE 2:** Comparison of different concentration of the O–methoxyamine HCl in pyridine (mg/ml) based on the repeatability of 75 MDMs detected in both types of samples, in the table was presented number of MDMs with RSD for plasma/serum ≤10%–30% and above 30%.

RSD (%)	15	20	25	30	35	40
	mg/mL					
	Number of MDMs (plasma/serum)					
≤ 10	2/25	16/36	20/53	39/42	30/52	25/7
≤ 20	1/29	24/20	24/12	18/27	20/8	22/24
≤ 30	2/8	8/6	9/3	7/2	7/8	7/18
> 30	70/13	27/13	22/7	11/4	18/7	21/26
Median RSD [%]	84.3/14.7	18.6/11.0	16.4/6.7	7.2/6.4	14.2/9.0	14.0/25.1

Subsequently, we tested five different MeOx volumes (10 μl, 20 μl, 30 μl, 40 μl, and 50 μl, all at 30 mg MeOx per mL pyridine concentration) (see Table 3) with the same volume of reagent for SIL (MSTFA with 1% TMCS), adjusted to 120 µl with heptane containing IS2. The TI of peaks area increased with the larger volume of tested reagent (Figure 3). We observed the highest TI for 30 and 50 µl for serum and plasma, respectively. The best repeatability (≤20% of RSD) for the majority of detected MDMs was obtained for the volume of 30 µl of the O–methoxyamine HCl in pyridine in both tested matrices. In plasma samples, the median RSD was much higher for 10 and 20 μl as compared to other values (32.5% for 10 μl, 29.2% for 20 μl, and 10.1%–12.6% for 30–50 μl). In serum samples, the median RSDs for all tested volumes were in the range of 5.0%–11.1%. Additionally, it was observed that lysine (Lys) and ascorbic acid were not detected while using the volume of 10 and 20 μl MeOx in both matrices, while 5–hydroxy–L–tryptophan was only detected in plasma samples, regardless the volume of MeOx. Due to the fact that reproducibility for volumes of 30 and 40 µl in serum was similar (RSDs for 68 and 65 metabolites ≤20% (RSD); median RSD 7.9 and 7.8%, respectively), the TI of all MDMs was the highest for 30 μl, for the next step of the optimization we chosen this volume of MeOx for both matrices.

**TABLE 3:** Comparison of different volume of the O–methoxyamine HCl in pyridine (µl) based on the repeatability of 75 MDMs detected in both types of samples, in the table was presented number of MDMs with RSD for plasma/serum ≤10%–30% and above 30%.

RSD (%)	10	20	30	40	50
	µl				
	Number of MDMs (plasma/serum)				
≤ 10	9/35	29/54	39/52	35/44	8/47
≤ 20	9/26	28/15	22/14	25/20	5/16
≤ 30	12/7	6/3	6/4	8/3	26/2
> 30	45/7	12/3	8/5	7/8	36/10
Median RSD [%]	32.5/11.1	29.2/5.0	12.6/7.9	10.1/7.8	30.4/8.8

In the next step we compared the effect of different conditions, i.e. time and temperature for MeOx process. We tested four common programs: 30 min at 37°C (P1), 1 h at 70°C (P2), 16 h at RT (P3) and 1 h at 70°C followed by 16 h at RT (P4). Obtained results are presented in Tables 4, 5. Repeatability for all metabolites was similar for all tested conditions in plasma samples, the RSDs for more than 70 MDMs were below 20%, while median RSD varied from 4.3 to 7.6%. In serum samples, we observed more differences in RSD values for tested conditions. The median RSDs were in the range from 9.4 to 18.5% for all tested programs. The RSD values were above 20% for more than 30 MDMs when the results for all tested programs were compared with an exception for MeOx process conducted for the program P3. The RSDs values were above 30% for 10 metabolites and the medium RSD for this condition was the lowest (9.4%). We observed differences in the intensities of the metabolites in tested matrices. The highest intensity for the majority of detected MDMs (53 from 75) was observed when program P3 was used in both matrices, the results obtained for program P2 were similar. In plasma samples, the lowest intensity was observed for more than 50 MDMs, whereas in serum samples for 48 MDMs, when the program P1 was used. In the case of valine (Val) and leucine (Leu) derivatisation, two chromatographic peaks were observed in plasma, namely Val 1TMS and Val 2TMS, Leu 1TMS, and2 TMS. The peak areas of mentioned metabolites were found to increase with longer reaction time. Similarly, derivatisation of glycine (Gly) was confirmed by the presence of two chromatographic peaks (Gly 2TMS and 3TMS). In this case, the peak area of Gly 3TMS was elevated with the increasing reaction time and temperature from 37°C, 30 min to 70°C, 1 h and finally 70°C 1h, 16 h (RT). However, in case of acetoacetate, which was confirmed by the presence of acetoacetate 2TMS and 3TMS, the intensity of 3TMS derivative decreased with longer reaction time. In serum samples we observed the same relation for AAs (Val, isoLeu, proline, Lys) and two CARBs, i.e. mannose and glucose. The intensity of the 3TMS derivative of these metabolites increased when temperature and time increased. We also observed that disaccharides were incompletely methoximated at 37°C for 30 min (P1), as we noticed significant peak broadening caused by multi peaks, which was not observed at 70°C.

**TABLE 4:** Comparison of different MeOx conditions based on the repeatability of 75 MDMs detected in both types of samples, in the table was presented number of MDMs with RSD for plasma/serum ≤10%–30% and above 30%.

RSD (%)	37°C, 30 min (P1)	70°C, 1 h (P2)	16 h, RT (P3)	1 h 70°C, 16 h RT (P4)
	Number of MDMs (plasma/serum)			
≤10	63/28	55/23	70/39	61/24
≤ 20	12/15	16/22	4/19	12/16
≤ 30	0/5	2/15	1/7	0/1
> 30	0/27	2/15	0/10	2/34
Median RSD [%]	4.5/15.9	7.6/18.5	4.3/9.4	5.7/17.5

**TABLE 5:** Comparison of different SIL conditions based on the repeatability of 75 MDMs detected in both types of samples, in the table was presented number of MDMs with RSD for plasma/serum ≤10%–30% and above 30%.

RSD (%) (Plasma/serum)	37°C, 30 min	37°C, 60 min	70°C, 30 min	70°C, 60 min
<5	38/25	37/17	41/24	37/38
<10	20/21	28/22	22/21	22/14
<15	8/14	7/10	7/13	8/10
<20	5/8	½	4/9	3/3
>20	4/7	2/24	1/8	5/10
Median RSD [%]	4.9/7.8	5.4/10.2	5.0/7.8	5.3/5.7

As the last optimization parameter, we tested the impact of different incubation temperatures and time of SIL process. Our first observation (based on Figure 3) was that the TI of all tested metabolites was higher in serum than in plasma, whereas when we compared the TI of MDMs in different conditions for both matrices, we observed that the TI was the highest for program: 70°C for 60 min, and the TI was comparable for the program performed at 37°C for 60 min. In serum samples, the median RSD was the highest (10.2%) when 37°C at 60 min program was used, the lowest median RSD (5.7%) was achieved when 70°C for 60 min program was tested. Additionally, this program was favorable for AAs and organic acids (OrgAs) for which repeatability of individual metabolites was the lowest. According to the results obtained for plasma samples we observed that a median RSDs were similar for all programs (4.9%–5.4%). Derivatisation of carboxylic acids, such as pyruvic, glyceric as well as lactic acid proceeded significantly better when 37°C for 30 min program was used, whereas derivatisation of the majority of AAs and FAs proceeded significantly better when 70°C for 60 min program was used. Additionally, at this conditions we did not observed the two chromatographic peaks for Ala and Val in both matrices, what proves that derivatisation was complete. Finally, due to the fact that comparable median RSDs were obtained for SIL at 70°C for 1 h for both matrices (5.3% and 5.7% for plasma and serum, respectively), we chose this program for the GC–MS analysis.

### Repeatability, reproducibility and stability of microbiota-dependent metabolites measurements

Including 4 h of equilibrium, the analysis of 50 replicates lasted about 35 h which is important as some of AAs are unstable after 36 h. In plasma samples, the repeatability test showed a median RSD of 12.6% ranging from 3.5% (lactic acid) to 37.3% ornithine (Orn). In serum samples, the repeatability test showed a median RSD from 4.6% (Thre) to above 50% (for 5–hydroxy–L–tryptophan, Orn and aspartic acid). In plasma samples, inter–batch median RSD across three batches was 23.5%, whereas in serum samples, inter–batch median RSD across three batches was 23.0% (Supplementary Table S2). Our method showed a good repeatability for compounds from different classes, including AAs, CARB and OrgAs. The repeatability over a long sequence (more than 50 injections at once) was in an acceptable range.

Obtained results showed that the majority of metabolites were stable even 48 h after the derivatisation with the median RSDs range between 8.8% and 12.8% in plasma and 6.6%–9.4% in serum. We observed that glutamine and benzoic acid were already unstable after 8 h. However, some of MDMs were observed as unstable after 36 h, e.g., two AAs and FAs in plasma and four AAs in serum samples. (Supplementary Table S3).

### Comparison results for both matrices

When we took into account intensity of individual metabolites (Supplementary Table S1), we observed that the intensities of 30 MDMs tested in our study were at a similar level in plasma and serum. For some metabolites we observed better reproducibility for plasma samples (see Supplementary Table S2). One third of measured metabolites had a higher intensity in plasma samples (e.g., AAs: Glu, Orn, Cre, GA, and glycine), whereas, the intensity of 22 metabolites was higher in serum samples mainly AAs (e.g., Met, aspartic acid and Cys). The total intensity of all tested metabolites was the higher in serum samples (Figure 3).

### Analysis of the clinical samples

**FIGURE 4:**
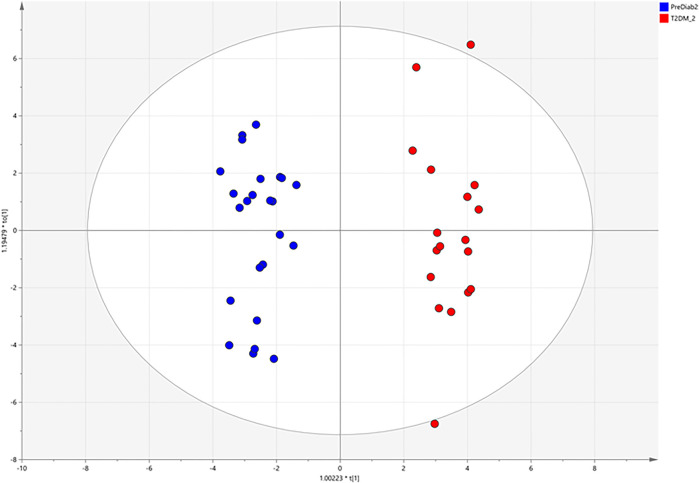
OPLS–DA score plots illustrating discrimination between the two studied groups based on obtained GC-MS data.

**FIGURE 5:**
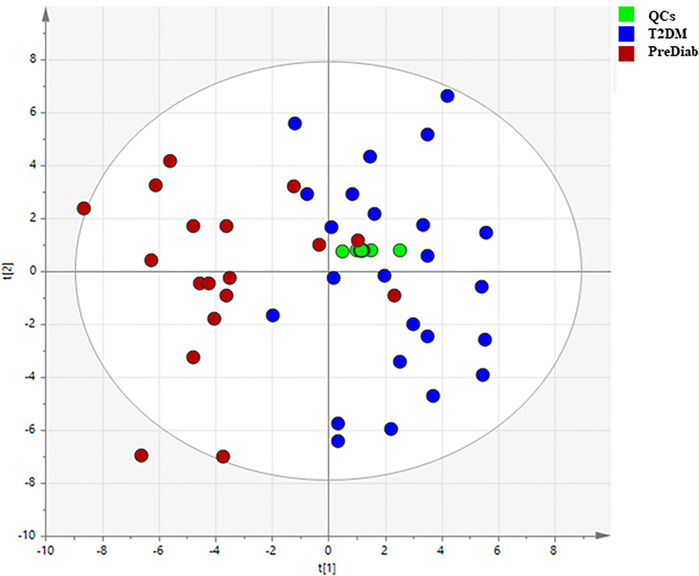
PCA plots illustrating classification of the two studied groups based on obtained GC–MS data.

Based on the above mentioned results, both matrices were suitable for the analysis of MDMs, but the observed intensity of most MDMs was relatively higher in serum samples. Due to this fact, we chose serum samples for further analysis. After data processing, 324 metabolic signals were detected. Signal grouping and filtering processes rendered a total of 275 metabolites from which a group of 98 metabolites could be identified taking into account several derivatives from one metabolite for some AAs and CARBs. RSD value for 89 metabolites was below 30% (80 MDMs) in the QC serum samples. To find metabolites discriminating the studied groups, OPLS–DA model was built and presented in Figure 4, additionally PCA plot was presented in Figure 5.

Our experiment revealed changes in 18 MDMs. These metabolites are mainly AAs, FAs, and CARBs (Table 6). In the OPLS–DA score plots, the PreDiab group and T2DM group were discriminated from each other with the model values of R_(cum)_^2^ = 0.912 and Q_(cum)_^2^ = 0.803. A cross–validation results using the “leave 1/3 out” approach showed that excluded samples were classified correctly in 94.5% ± 5.2%. Among the statistically significant metabolites, alpha–hydroxybutyric acid (α–HBA) (FC = 1.24), Leu (FC = 1.28), Glu (FC = 1.48), tryptophan (Trp) (FC = 1.96), Cys (2.07) and stearic acid (SA) (FC = 1.79) were increased, while Cre (FC = 0.76), glutamic acid (GA) (FC = 0.65) and (Orn) (FC = 0.63) decreased in T2DM compared to prediabetic patients.

**TABLE 6:** Statistically significant changes for MDMs detected in serum. Metabolites checked in the Human Metabolome Database (HMDB) (http://www.hmdb.ca access: 20^th^ April 2022); rt, retention time (minutes); p (corr)—predictive loading values in the OPLS-DA, VIP—variable importance in projection; CV, coefficient of variation of the metabolites in the QC samples; FC, fold change in the comparison (PreDiab vs. T2DM).

Metabolites	HMDB	rt	p (corr)	VIP	FC	CV in QC [%]
α–hydroxybutyric acid	HMDB00008	7.8	0.63	1.38	1.24	3.9
Creatinine	HMDB00562	13.5	–0.44	1.33	0.76	17.1
Cystine	HMDB00192	20.7	0.39	1.89	2.07	15
Galactonic acid	HMDB00565	18.3	–0.42	1.48	0.7	19.1
Gluconic acid	HMDB00625	18.3	–0.42	1.6	0.67	20.2
Glutamic acid	HMDB00148	13.2	–0.73	2.04	0.65	9.9
Glutamine	HMDB00641	13.2	0.43	1.62	1.48	26
Glycerol 1–phosphate	HMDB00126	15.8	–0.4	1.07	0.82	12.8
Kynurenine	HMDB00684	20.1	–0.48	1.17	0.8	15
Leucine	HMDB00687	10.1	0.69	1.58	1.28	9.8
Malic acid	HMDB31518	12.6	–0.44	1.19	0.79	7
Mannose	HMDB00169	17.4	0.67	1.19	1.17	27
Oleic acid	HMDB00207	20.4	0.47	1.42	1.29	5.2
Ornithine	HMDB00214	16.4	–0.55	1.75	0.63	12.5
Serotonin	HMDB00259	22.4	0.53	1.9	0.45	20.8
Stearic acid	HMDB00827	20.6	0.91	2.69	1.79	10
Trans–4–hydroxy–L–proline	HMDB00725	13.1	0.52	1.63	0.56	19.7
Tryptophan	HMDB00929	20.3	0.67	2.75	1.96	26.3

**FIGURE 6:**
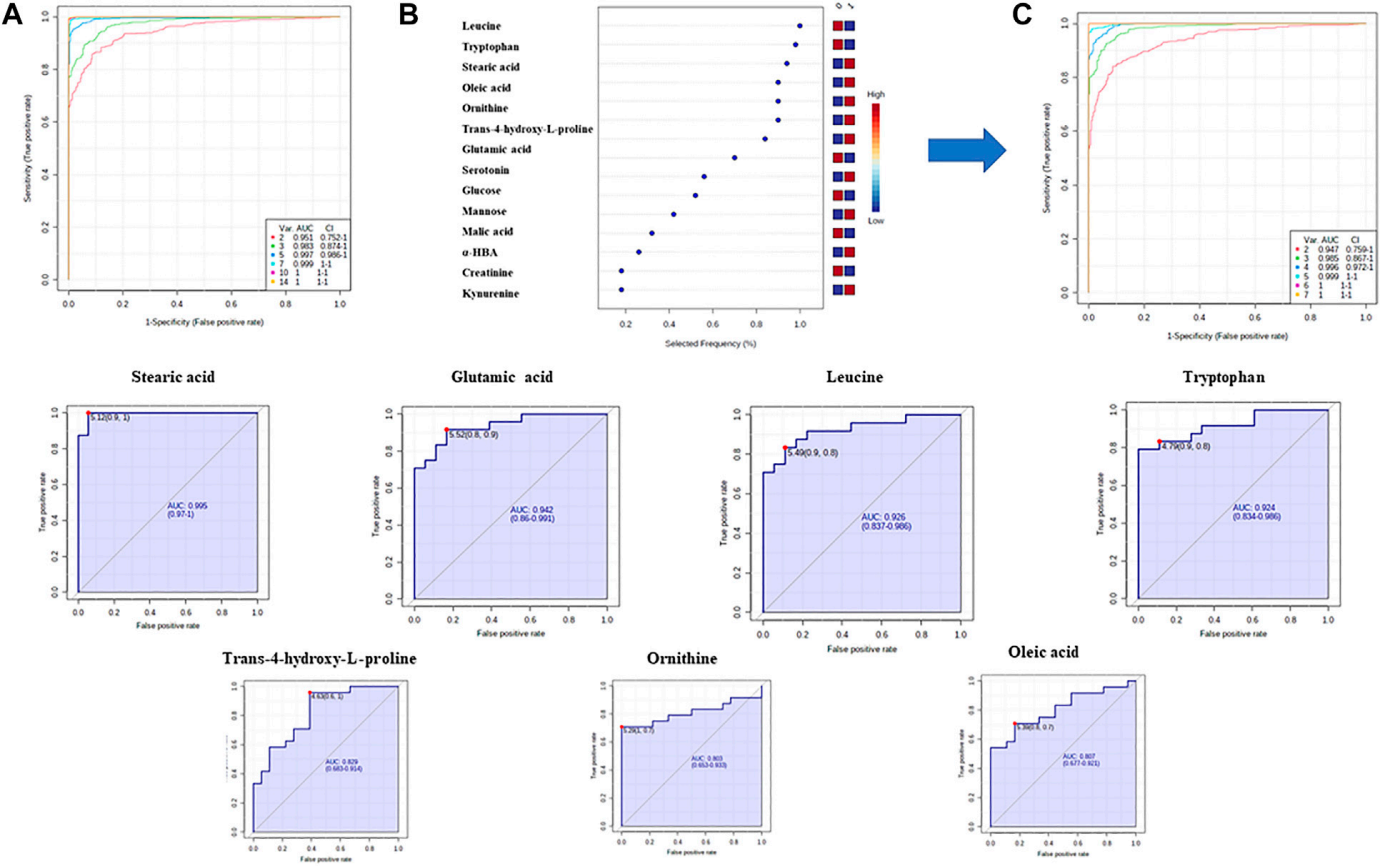
Discovery of a potential biomarker panel in T2DM by GC–MS untargeted metabolomics. **(A)** ROC curves and AUC values based on SVM classification model for all statistically significant metabolites; **(B)**—plot of the most important and frequently selected variables during the panel exploration analysis. **(C)**—ROC curves and AUC values based on SVM classification model for seven metabolites.

Finally, in order to evaluate a potential of significant metabolites to serve as biomarkers indicating T2DM development in prediabetic patients, a multivariate receiver operating characteristic (ROC) curves were obtained. ROC analysis based on support vector machine (SVM) modeling was employed to perform the automatic selection of the best metabolites combination. ROC curves were constructed for the selected 14 MDMs (Figure 6A) using the relative metabolite contents of the experimental groups. For seven MDMs (out of 14) showing the best discriminatory power (Figure 6B) individual ROC curves were built (Figure 6C).

## Discussion

### Optimization of the sample preparation step

Recent studies have indicated that GM are associated with various metabolic diseases (Tanase et al., 2020). Most studies used fecal samples as they can directly reflect the human GM composition (Mojsak et al., 2022). However, an increasing number of studies (Visconti et al., 2019) have revealed that although the concentration of GM related metabolites in circulation is far lower than that in feces, these metabolites play an important role in modulating metabolism. Therefore, it is essential to develop a comprehensive and efficient method for analyzing MDMs in biofluid samples. Based on the literature review, it was noticed that several protocols for the biofluid sample preparation for metabolomics study based on GC–MS are described (Drogan et al., 2015; Fiehn, 2016; Liu et al., 2017; Org et al., 2017; He et al., 2021; Raczkowska et al., 2021), differing in the type of solvent or derivatisation procedures used. Therefore, we chose the most frequently used solvents and variables at the derivatisation stage, then we optimized them in the context of the MDMs in two matrices.

### Selection of extraction solvents

Solvents are known to be one of the primary factors that affect the number, type, and abundance of endogenous metabolites detected in biological samples (He et al., 2021). The most widely used protocol for global metabolomics is protein precipitation with solvent using a plasma/serum–to–solvent ratio of 1–3 or 4. Cold solvent is added to minimize the extent of enzymatic conversion of metabolites and to improve protein precipitation (Sitnikov et al., 2016). Therefore, in the first step of this study, we screened the effect of five solvent variants, widely used in GC–MS–based metabolomics. To the best of our knowledge, studies comparing this type of solvent mix are still lacking. Sitnikov et al. (2016) compared seven different extraction solvents but for LC–MS–based metabolomics. Extraction using MeOH:EtOH, and MeOH demonstrated the best repeatability in comparison to all other methods regardless the LC–MS method employed. In our study, we also observed the best results for MeOH but with the addition of H_2_O in a ratio of 9:1. In the previous study presented by Jiye et al. (2005) (Trygg et al., 2005), the use of MeOH:H_2_O mixture (v/v; 8:1) in GC–MS–based metabolomics analysis of plasma samples provided optimal results in terms of completeness, efficiency, and reproducibility of extraction in comparison to other tested solvents (EtOH, ACN, acetone, chloroform), which is consistent with our findings. According to Can Eylem and coworkers (Eylem et al., 2022), who studied the effect of five different solvents (acetone, ACN, EtOH, MeOH, and H_2_O), the addition of water in the MeOH extraction had a positive effect on the extraction of polar metabolites, resulting in higher peak areas.

### Optimization of methoxymation and silylation conditions

Based on the literature review (Moros et al., 2017; Miyagawa and Bamba, 2019; Fritsche-Guenther et al., 2021), it was observed that many factors at both stages (MeOx and SIL) contribute to the reaction speed and completeness of derivatisation reaction which may directly impact the repeatability and reproducibility of sample analyses. Due to the degradation of metabolites, it is difficult to obtain good repeatability during the batch derivatisation, as the time between completing the derivatisation process and GC analysis differ from sample to sample (Miyagawa and Bamba, 2019). Despite this fact, several attempts (Moros et al., 2017; Miyagawa and Bamba, 2019; Fritsche-Guenther et al., 2021) have been made to improve sample preparation procedure for GC–MS metabolomics. In the research presented by Fritsche–Guanter and coworkers (Fritsche-Guenther et al., 2021), different derivatisation conditions were taken into account during optimization of the protocol for fully automated and effective protein precipitation and extraction of 42 metabolites from plasma, serum and liver samples. It is known that results for manual and fully automated methods differs, as presented by Zarate et al. (2016). In other studies, researchers (Moros et al., 2017; Miyagawa and Bamba, 2019; Eylem et al., 2022) observed differences in response and repeatability of metabolites due to the change of the parameters of both derivatisation steps, and for this reason we optimized method at both stages.

In order to reduce the number of derivatisation products, MeOx–derivatisation was employed before TMS–derivatisation. Different MeOx concentration–volume combinations were reported for derivatisation of biofluid samples. In most protocols (Moon et al., 2013; Org et al., 2017; Gar et al., 2018; Sobczak et al., 2019; He et al., 2020; Mojsak et al., 2021), MeOx in the volume of 50–125 μl and the concentration of 15–40 mg/ml is added to dried extract. Based on the literature findings (Moros et al., 2017; Miyagawa and Bamba, 2019; Engel et al., 2020; He et al., 2020; Fritsche-Guenther et al., 2021; He et al., 2021), there is information on the effect of the MeOx concentration on the derivatisation process. The concentration of MeOx reagent most frequently reported in the literature is 20 mg/ml. In the research presented by Eylem and coworkers (Eylem et al., 2022), a high correlation between the response and MeOx concentration was found, but details were not shown. For optimization, authors used the concentration of 30 mg/ml, which is consistent with our study, although the volume of MeOx was different. We chose the concentration of 30 mg/ml due to better repeatability for both matrices and relatively high intensity for most of the studied metabolites. In the study presented by Fritsche–Guenter (Fritsche-Guenther et al., 2021), but in fully automated method of optimization, it was observed that the overall areas of metabolites decreased around 30% with increasing concentration of MeOx.

When we optimized a volume of O–methoxyamine HCl solution in pyridine, the volume of MSTFA with 1% TMCS was also changed to be equal to the volume of O–methoxyamine HCl solution in pyridine. Such approach is used in many GC-MS-based metabolomics studies on plasma or serum samples (Moros et al., 2017; Raczkowska et al., 2021). On the other hand, it was shown that efficient derivatisation can be obtained by decreasing the amount of MeOx and increasing the amount of SIL reagent (Fiehn, 2016). According to Miyagawa and Bamba (Miyagawa and Bamba, 2019), who presented the comparison of the results obtained with the use of various volumes of O–methoxyamine HCl solution in pyridine, and MSTFA, it was revealed that the peak areas of the number of metabolite derivatives increased (mainly AAs) with the increase of the volume of both reagents employed for the derivatisation, which is consistent with our study. Bekele and coworkers (Bekele et al., 2014) found a similar association between the volume of MSTFA and the number of detected peaks.

Based on the literature review, the degree of completion of the both MeOx and SIL reaction depends on reaction time and temperature (Miyagawa and Bamba, 2019) and a lot of attention has been paid to this step (Miyagawa and Bamba, 2019; He et al., 2020). The most commonly accepted MeOx conditions include either reaction in high temperature for short time or low temperature for prolonged time. On the other hand, incubation process provides the completion of MeOx; however, it could also result in progressive degradation of heat labile metabolites (Bekele et al., 2014). To maximize coverage of metabolites in reproducible way, while minimizing chemical and physical degradation, compromises are inevitable. The results obtained with different temperature conditions differed depending on a particular metabolite, and in terms of different classes of metabolites some trends were noticed. Higher MeOx temperature is considered to enhance derivatisation efficiency by increasing solubility of metabolites. Bekele and coworkers (Bekele et al., 2014) reported that long term, low temperature MeOx was favorable for organic acid and AAs, while either long term low temperature or a short term high temperature MeOx was favorable for α–keto acids. According to Miyagawa and Bamba (Miyagawa and Bamba, 2019), disaccharides were incompletely methoxymated at 37°C for 30 min which resulted in a significant peak broadening caused by multipeaks, which was also observed in the presented study. Shepherd et al. (Shepherd et al., 2007) reported that CARB (glucose and fructose) were partially methoxymated at 30°C for 45 min and sucrose was hydrolyzed to glucose and fructose at 100°C when the process was longer than 45 min. MeOx results at 37°C for minimum 60 min were better in terms of glucose and maltose detection (Miyagawa and Bamba, 2019). Sterols are often derivatized at 60–100°C, but Miyagawa and Bamba observed transformation of these metabolites at 70°C (Miyagawa and Bamba, 2019). Overall, repeatability was better for all of the individual classes when incubation protocol was performed at 60°C for 30 min. According to Pasikanti et al. (Pasikanti et al., 2008), MeOx must be performed over a relatively long period of time (up to 17 h) and/or at high temperature to provide complete derivatisation. The same as in the study presented by Musharraf et al. (Musharraf et al., 2013), in which MeOx (16 h) was found to be the best in terms of the number of metabolites, which is consistent with our findings.

Finally, we compared the conditions for the last step of sample preparation—SIL. This is a classical derivatisation method employed to introduce a silyl group to a metabolite by replacing active hydrogen atoms (of carboxyl groups, amino and hydroxyl groups) to generate stable, more volatile and less polar metabolites. Several derivatisation reagents have been applied for the derivatisation of endogenous metabolites. The most popular reagents are MSTFA and BSTFA (N,O-Bis (Trimethylsilyl) trifluoroacetamide), with or without the catalyst, 1% (TMCS). Moros et al. (2017) demonstrated that MSTFA with 1% TMCS was found to provide more repeatable results and enable the detection of more derivatives compared to BSTFA with 1% TMCS in plasma extracts. In the previous study, Fiehn (2016) also confirmed that MSTFA was superior over BSTFA in regard to the completeness of NH silylation of AAs and amines, and for this reason we did not optimize SIL reagent and chose MSTFA with 1% TMCS for optimization of other derivatisation conditions.

Factors like temperature and time of SIL contribute to the reaction speed and completeness of SIL process. Namely, differences in metabolite peak areas of various derivatives were observed in different biological matrices as a result of changing the SIL time and temperature (Eylem et al., 2022). The most frequently reported temperature and duration of SIL for both matrices range between 30 and 70°C and 30–120 min (Danielsson et al., 2012; Fiehn, 2016; Moros et al., 2017; Kiseleva et al., 2021). Therefore, we compared SIL conditions (37°C and 70°C for 30 and 60 min, respectively) the most commonly used in the metabolomics studies. Long term and high temperature SIL was favorable for FAs and CARB. On the other hand, this conditions had negligible effect on OrgAs and AAs. It is difficult to adapt a unique SIL process at which all functional groups would be derivatized because reaction kinetics differ among functional groups. The ideal scenario would be if SIL of every functional group of metabolites was completed at the selection time. The completion of reaction without any degradation effect for all metabolites remains a challenge. It was proved that metabolites containing–OH, –COOH and ketone groups were derivatized 5 h after the addition of MSTFA whereas the derivatisation of–NH2 groups was still progressing after a day, at which point, other products began to degrade (Moldoveanu and David, 2018).

Multiple peaks are usually produced when this step of derivatisation is incomplete. At 70°C for 60 min, we did not observe multiple peaks for AAs such as Ala and Val in neither of matrices, thus we assumed that a SIL process was completed. Danielsson et al. (2012) observed, similarly to our findings, that long incubation time is needed to complete a derivatisation process. Furthermore, these results imply that long SIL periods (>30 min) are needed to provide effective derivatisation of slowly reacting metabolites. Two peaks were found from the TMS derivatisation of sugars (e.g., glucose and mannose), which was in accordance to the previously published literature (Villas-Bôas et al., 2011; Zarate et al., 2016). Finally, we chose 16 h at RT for methoxymation and 1 h at 70°C for SIL. It was proved in the literature that a longer MeOx at low temperature in combination with a high SIL temperature provide a similar results to a short–time methoximation at a high temperature in combination with a low SIL temperature (Bekele et al., 2014), which is consistent with our findings. In previously reported study it was found that MeOx (16 h) and SIL (1 h) provide the best results in terms of the number of metabolites (Musharraf et al., 2013).

### Results of the comparison between both tested matrices

There are metabolomics studies showing the differences between serum and plasma metabolic profiles (Kaluarachchi et al., 2018). Depending on the type, class or particular metabolite, one matrix can be better than the other to perform metabolomics analyses. In general, serum is favored due to the slightly higher metabolites concentration compared to plasma (Yu et al., 2011), which is consistent with our results taking into account TI (Figure 3). Deproteinization of serum eliminates the volume fraction of proteins and distributes the remaining small molecular weight constituents in a smaller volume, thus making them more concentrated. On the other hand, there are studies confirming that the level of some metabolites are lower in serum compared to EDTA plasma (Suarez-Diez et al., 2017; Sotelo-Orozco et al., 2021). It is obvious that serum and plasma are similar in terms of some characteristics and different in other, and these differences may be important for bio specimen selection and metabolite identification in metabolic phenotyping studies. Due to all the above–mentioned factors, we have made the effort to perform a comprehensive optimization for 75 MDMs in both matrices**.** As far as we know, no comprehensively optimized method, including the impact of most commonly used mixtures of solvents and derivatisation conditions, for profiling of both plasma and serum metabolites as MSTFA derivatives has been presented thus far. The most considerable differences concern AAs, associated with GM and T2DM development thus, the optimization of the methods for both matrices is crucial. It has been reported in previous optimization study (Moros et al., 2017), that good repeatability, particularly for AAs is difficult to achieve during batch derivatisation, as the time between the completion of derivatisation reaction and GC analysis differs from sample to sample. Optimisation of repeatable and reproducible method for the quantification of MDMs was essential because we applied this method for the analysis of clinical serum samples to evaluate changes in MDMs related to T2DM development.

## Analysis of clinical samples

Many changes in gut composition have been reported in T2DM patients and it was proved that GM may be a significant environmental factor involved in the onset and progression of T2DM (Tanase et al., 2020). Larsen et al. (2010) conducted one of the first studies in humans comparing the GM between individuals with T2D and healthy controls. This study demonstrated that the phylum *Firmicutes* and the class *Clostridia* were less abundant in the T2DM group compared to the control group, whereas the class *Betaproteobacteria* was more abundant in T2DM subjects and positively correlated with plasma glucose level. In the same study (Pedersen et al., 2016) it was shown that dysbiosis of GM impacts serum metabolome and contributes to IR. Therefore, analysis of serum metabolome can be potentially used for T2DM diagnosis, also in the context of MDMs. Based on the literature review we proved that a variety of biological markers detected in biofluids by GC-MS (Gar et al., 2018; Adamska-Patruno et al., 2019; Mojsak et al., 2021) are associated with T2DM. GC–MS–based metabolomics is a powerful approach for studying pathophysiological processes (Raczkowska et al., 2021), and has been used to identify complex endogenous metabolic phenotypes in various diseases (Zhang et al., 2020). For these reasons GC–MS was used to identify the differences in serum samples between diabetic and prediabetic subjects.

Several studies have indicated the influence of bacterial taxa on lipid and FA levels in blood samples (Rooks and Garrett, 2016; Org et al., 2017). The serum FAs profile is determined by host endogenous FA metabolism (Baylin and Campos, 2006). Data presented by Org and coworkers (Org et al., 2017) showed that fasting serum monounsaturated and saturated FAs are strongly associated with an increased abundance of *Blautia* and *Dorea* and decreased abundance of *Coprococcus* and *Peptococcaceae.* As it can be seen in Table 6, we indicated statistically significant changes in FAs (oleic acid (OA) and SA). Studies using metabolic profiling support the importance of FAs in the prediction of T2DM onset. It was confirmed that FA levels were significantly higher in newly diagnosed (Lu et al., 2016; Castro-Correia et al., 2017) and long–term monitored patients (Spiller et al., 2018) with T2DM, what is in line with the results of our research. Among all metabolites used for ROC analysis, SA exhibited the highest AUC value (0.995) (Figure 6). An increased concentration of serum SA plays a fundamental role in the development of beta cell dysfunction and T2DM, as this FA is the major contributor to lipotoxicity in beta cells (Vilas-Boas et al., 2021). It was consistent with the findings of Lu et al. (2016) and Zhao et al. (2017), who identified FAs as the most important pathogenic factors for insulin resistance and T2DM.

Lipid oxidation may cause an increase of α–HB level, and we indicated elevated level (FC = 1.24) of this metabolite in T2DM group. It was confirmed that a high level of α–HB is common for T2DM, and it was previously identified as a marker and predictor of T2DM (Gall et al., 2010; Cobb et al., 2016; Vangipurapu et al., 2019; Ferrannini et al., 2022). α–HBA is a byproduct of α–ketobutyric acid synthesis, a product of AA catabolism (Thre and Met) and glutathione anabolism (Cys formation pathway) in hepatic tissue (Sousa et al., 2021). Trico and coworkers (Tricò et al., 2017) has demonstrated that α–HBA level increased in IR, potentially due to a metabolic overload (through BCAA and free FAs) and oxidative stress (by the higher intracellular NADH/NAD + ratio).

GM can promote the production and utilization of AAs, which can be absorbed across the gut and accumulate in the blood. Thus, the GM could influence serum AA levels (Shen et al., 2021) (Supplementary Table S1). Subjects with IR also exhibit proliferation of *Prevotella copri* and *Bacteroides vulgatus*, which elevate the circulating levels of BCAAs (Li et al., 2017). AAs have an important role in multiple pathophysiological processes (Zhou et al., 2021). Disturbances in AA metabolism are closely involved in the pathogenesis of T2DM. Several AAs, including especially BCAAs and aromatic AAs, have been shown to be associated with increased risk of T2DM (Vangipurapu et al., 2019; Long et al., 2020). Pedersen et al. (2016) suggested that intestinal microbiota could be an important source of increased levels of BCAAs and play a key role in insulin resistance. In the presented study, in terms of BCAA, differences in the level of Leu were observed. Leu serum abundance was statistically higher in T2DM than in prediabetic patients. Our results are in accordance with the other findings showing significant association of BCAAs (especially Leu) with T2DM (Lynch and Adams, 2014; Flores-Guerrero et al., 2018; Gar et al., 2018; Holeček, 2018).

Elevation of BCAAs leads to accumulation of carnitines in muscle, which induces oxidative stress and mitochondrial dysfunction, thereby aggravates insulin sensitivity (Lynch and Adams, 2014). As it was already shown (Hu et al., 2019; Mojsak et al., 2021), lower level of Cre in serum, also observed in our study, might reflect a lower amount of skeletal muscle thus fewer target sites for insulin which may partially explain the pathogenesis of T2DM associated with lower serum level of Cre. In the comparison of PreDiab vs. T2DM_group, we also observed decreased level of this metabolite in T2DM group. In the study presented by Agus and coworkers (Agus et al., 2018), bacteria and fungi capable of degrading Cre have been identified in the human colon. Consequently, accurate determination of these metabolites can be essential for the early diagnosis of T2DM.

Previous studies have suggested that several metabolites of the kynurenine pathway are diabetogenic to humans, which is directly or indirectly controlled by microbiota (Oxenkrug, 2013). Kynurenine pathway, involved in Trp metabolism, was previously reported as being upregulated in T2DM. Enzyme responsible for conversion of Trp to kynurenine has been shown to be regulated by microbiota (Agus et al., 2018). In our study, we observed statistically significant changes in an essential AA—Trp (FC = 1.96) in prediabetic patients in comparison to T2DM. Trp is also a precursor for serotonin synthesis in the gut mucosa (Jenkins et al., 2016; Gao et al., 2020). Interestingly, in our study, we observed elevated level of serotine, associated with the increased risk of T2DM which is consistent with previous findings (Yabut et al., 2019).

Org and coworkers (Org et al., 2017) showed the connection between GM and Glu levels. Higher Glu levels in plasma were significantly associated with higher bacterial abundance such as *Clostridales*. Several species from unclassified *Clostridales* positively correlated with glutamine concentrations. It was revealed that Glu and GA are related to T2DM. In β–cells, glutamine is transported by blood and accumulated in plasma, then further converted to GA (Jenstad and Chaudhry, 2013). Cheng et al. reported that plasma Glu, glutamate, and the Glu/glutamate ratio were strongly associated with IR. GA concentration is also one of the most important indicators of diabetic retinopathy (Cheng et al., 2012).

To evaluate the utility of significant metabolites as potential T2DM biomarkers, ROC curve analyses were performed (Figure 6). The AUC of the combination of five AAs and two FAs revealed the most powerful capability to discriminate between prediabetics and T2DM patients. This combination might serve as a potential T2DM indicator as it provides improved specificity compared to single metabolite measurement. The diagnostic accuracy of these metabolites could be further enhanced by combining it with other routine diagnostic parameters such as FP glucose or HbA1c.

## Conclusion

In the presented study, we focused on the comparison of different sample preparation conditions including different solvents for protein precipitation and two steps of derivatisation, MeOx followed by SIL. We analysed two matrices (serum and plasma) in the context of 75 MDMs. The comprehensive evaluation of the results revealed that sample preparation with methanol with the addition of water provided the most stable signals for MDMs. Our results also suggest that a MeOx volume and concentration has the greatest impact on repeatability and intensity, whereas derivatisation reaction conditions mostly influence the reaction speed and completeness of this process.

It is important to have a repeatable and reproducible method for the determination of MDMs in blood-related samples, as it can be used to study the role of such a metabolites in the development of different diseases. In this study we applied this method to analyze serum samples obtained from prediabetic and T2DM patients. In total, 18 MDMs discriminated T2DM patients from prediabetics. Seven of them (SA, GA, Leu, Trp, Trans–4–hydroxy–L–proline, Orn and OA), based on the SVM classification model, were selected as a panel of potential biomarkers, able to distinguish between patients with T2DM and prediabetes. As our findings were derived from a small group, future validation of these results in a large–scale cohort study is needed.

## Data Availability

The raw data supporting the conclusion of this article will be available upon request in justified cases.

## Supplementary material

SUPPLEMENTARY TABLE S1 List of MDMs considered for optimization. rt, retention time; HMDB- Human Metabolome Database; T, target ion; QI 1, first qualifier ion; QI 2, second qualifier ion; RI, retention index. All compounds reported in the table are three methyl silylated (TMS), and the following number refers to the number of active hydrogen replaced with the TMS group, as reported in the Fiehn library; *confirmation in the literature that metabolite may be related to the GM or that metabolite may be part of the pathway that is disturbed by GM)

SUPPLEMENTARY TABLE S2 Repeatability and reproducibility of MDM measurments.

SUPPLEMENTARY TABLE S3 Stability of MDMs in both matrices.
