# Supplementary material for: Optimization of a GC-MS method for the profiling of microbiota-dependent metabolites in blood samples: An application to type 2 diabetes and prediabetes
Source: Front Mol Biosci. 2022 Sep 23;9:982672. doi: 10.3389/fmolb.2022.982672 (PMC9538375; doi:10.3389/fmolb.2022.982672)
Supplement: Supplementary file 1 [file Table1.docx]

**Table S1.** List of MDMs considered for optimization. **(**rt, retention time; HMDB- Human Metabolome Database; T, target ion; QI 1, first qualifier ion; QI 2, second qualifier ion; RI, retention index. All compounds reported in the table are three methyl silylated (TMS), and the following number refers to the number of active hydrogen replaced with the TMS group, as reported in the Fiehn library; *confirmation in the literature that metabolite may be related to the GM or that metabolite may be part of the pathway that is disturbed by GM).

| **Metabolites** | **Group of metabolites** | **HMBD** | **RT** | **TI** | **QI 1** | **QII 2** | **RI** | ***References** |
| --- | --- | --- | --- | --- | --- | --- | --- | --- |
| Pyruvic acid | Alpha-keto acids and derivatives | HMDB00243 | 6.5 | 174 | 89 | 59 | 720 | (95) |
| Lactic acid | Alpha hydroxy acids and derivatives | HMDB00190 | 6.6 | 147 | 73 | 117 | 732 | (40) |
| Glycolic acid | Alpha hydroxy acids and derivatives | HMDB00115 | 6.9 | 147 | 73 | 66 | 745 | (40) |
| Valine 1 | AAs, peptides, and analogues | HMDB00883 | 7.1 | 72 | 55 | 75 | 763 | (21, 38) |
| Valine 2 |  |  | 8.9 | 144 | 218 | 73 | 898 |  |
| Alanine 1 | AAs, peptides, and analogues | HMDB00161 | 7.3 | 116 | 73 | 147 | 774 | (21, 37), |
| Acetoacetate 1 | Short-chain keto acids and derivatives | HMDB0304256 | 7.4 | 89 | 59 | 186 | 785 | (11, 21) |
| Acetoacetate 2 |  |  | 7.7 | 73 | 89 | 202 | 807 |  |
| 3-methyl-2-oxobutanoic acid | FA esters | HMDB30027 | 7.5 | 89 | 186 | 202 | 806 | (21) |
| Glycine 1 | AAs, peptides, and analogues | HMDB00123 | 7.5 | 102 | 147 | 73 | 985 | (21) |
| Glycine 3 |  |  | 10.1 | 174 | 248 | 147 | 1310 |  |
| α-Hydroxybutyric acid | Alpha hydroxy acids and derivatives | HMDB00008 | 7.6 | 131 | 147 | 131 | 773 | (21, 96), |
| Oxalic acid | Dicarboxylic acids and derivatives | HMDB02329 | 7.9 | 190 | 147 | 190 | 806 | (97) |
| p-cresol | Cresols | HMDB01858 | 8.1 | 165 | 180 | 91 | 828 | (21) |
| β-Hydroxybutyric acid | Beta hydroxy acids and derivatives | HMDB00011 | 8.1 | 147 | 73 | 117 | 815 | (21) |
| Leucine 1 | AAs, peptides, and analogues | HMDB00687 | 8.2 | 86 | 75 | 73 | 832 | (11, 38) |
| Leucine 2 |  |  | 9.9 | 158 | 73 | 147 | 948 |  |
| Isolucine 1 | AAs, peptides, and analogues | HMDB00172 | 8.2 | 86 | 73 | 69 | 853 | (38) |
| Isolucine 2 |  |  | 9.9 | 158 | 73 | 218 | 965 |  |
| Methylalanine | AAs, peptides, and analogues | HMDB94692 | 8.3 | 130 | 73 | 147 | 844 | (21) |
| Proline 1 | AAs, peptides, and analogues | HMDB00162 | 8.4 | 70 | 75 | 103 | 850 | *(21)* |
| Proline 2 |  |  | 9.9 | 142 | 73 | 216 | 975 |  |
| 2-ketoisocaproic acid 1 | Short-chain keto acids and derivatives | HMDB00695 | 8.2 | 189 | 89 | 189 | 851 | *(38)* |
| 2-ketoisocaproic acid 2 |  |  | 8.9 | 200 | 216 | 189 | 886 |  |
| Urea | Ureas | HMDB00294 | 9.4 | 147 | 189 | 73 | 924 | *(21)* |
| Benzoic Acid | Benzoic acids and derivatives | HMDB01870 | 9.5 | 179 | 105 | 135 | 935 | *(98)* |
| Ethanolamine | Amines | HMDB00149 | 9.7 | 174 | 174 | 174 | 941 | (21) |
| Glycerol | CARBs and CARB conjugates | HMDB00131 | 9.8 | 205 | 147 | 205 | 950 | *(21)* |
| Phosphoric acid | Non-metal phosphates | HMDB01429 | 9.8 | 299 | 73 | 314 | 951 | *(99)* |
| Succinic acid | Dicarboxylic acids and derivatives | HMDB00254 | 10.4 | 247 | 73 | 75 | 990 | *(40)* |
| Glyceric acid | CARBs and CARB conjugates | HMDB00139 | 10.6 | 189 | 147 | 189 | 1008 | *(100)* |
| Fumaric acid | Dicarboxylic acids and derivatives | HMDB00134 | 10.9 | 245 | 147 | 73 | 1025 | *(35)* |
| Serine | AAs, peptides, and analogues | HMDB00187 | 11.0 | 204 | 73 | 218 | 1040 | (21, 37), |
| Nonanoic acid | FAs and conjugates | HMDB00847 | 11 | 215 | 215 | 215 | 1019 | *(42)* |
| Threonine | AAs, peptides, and analogues | HMDB00167 | 11.3 | 218 | 117 | 291 | 1064 | *(21)* |
| Aminomalonic acid | AAs, peptides, and analogues | HMDB01147 | 12.4 | 218 | 147 | 218 | - | (21, 35) |
| Aspartic acid | AAs, peptides, and analogues | HMDB00191 | 12.5 | 160 | 130 | 117 | 1103 | *(37)* |
| Malic acid | Beta hydroxy acids and derivatives | HMDB31518 | 12.7 | 233 | 133 | 245 | 1164 | *(35)* |
| Threitol | CARB and CARB conjugates | HMDB04136 | 12.9 | 217 | 147 | 217 | 1177 | *(101)* |
| Methionine | AAs, peptides, and analogues | HMDB00696 | 13.1 | 176 | 73 | 128 | 1195 | *(21)* |
| 5-Oxoproline/ Pyroglutamic acid | AAs, peptides, and analogues | HMDB00267 | 13.1 | 156 | 73 | 147 | 1196 | (21) |
| Trans-4-hydroxy-L-proline | AAs, peptides, and analogues | HMDB00725 | 13.2 | 230 | 73 | 140 | 1202 | *(21)* |
| Iminodiacetic acid | AAs, peptides, and analogues | HMDB11753 | 13.4 | 232 | 73 | 147 | 1206 | *(21)* |
| Glutamic acid | AAs, peptides, and analogues | HMDB00148 | 13.3 | 246 | 73 | 128 | 1298 | *(37)* |
| Phenylalanine 1 | AAs, peptides, and analogues | HMDB00159 | 13.5 | 120 | 146 | 73 | 1226 | *(21)* |
| Phenylalanine 2 |  |  | 14.4 | 218 | 192 | 73 | 1303 |  |
| 2-Aminoadipic | AAs, peptides, and analogues | HMDB0302754 | 13.4 | 205 | 131 | 103 | *1230* | *(21)* |
| Cysteine | AAs, peptides, and analogues | HMDB00574 | 13.5 | 218 | 220 | 73 | 1231 | *(21)* |
| Creatinine | AAs, peptides, and analogues | HMDB00562 | 13.5 | 115 | 73 | 100 | 1233 | *(21)* |
| Threonic acid | CARBs and CARB conjugates | HMDB00943 | 13.6 | 292 | 205 | 220 | 1235 | *(21)* |
| Ketoglutaric acid | Keto acids and derivatives | HMDB00208 | 13.9 | 198 | 147 | 75 | 1250 | *(40)* |
| Glutamic acid | AAs, peptides, and analogues | HMDB00148 | 14.3 | 246 | 73 | 128 | 1298 | *(37)* |
| Pyrophosphate | Non-metal pyrophosphates | HMDB00250 | 14.7 | 451 | 73 | 466 | 1346 | *(21)* |
| Lauric acid | Organosulfonic acids and derivatives/ | HMDB00251/ HMDB00638 | 14.8 | 257 | 73 | 117 | 1329 | *(21)* |
| Asparagine | AAs, peptides, and analogues | HMDB0251512 | 14.9 | 116 | 73 | 231 | 1346 | *(37)* |
| Lysine 2 | AAs, peptides, and analogues | HMDB00182 | 17.8 | 317 | 73 | 317 | 1596 | (21) |
| Glutamine | AAs, peptides, and analogues | HMDB00641 | 16.1 | 245 | 156 | 73 | 1432 | (21), |
| Ornithine | AAs, peptides, and analogues | HMDB00214 | 16.4 | 142 | 73 | 174 | 1496 | *(21)* |
| Hypoxanthine | Purines and purine derivatives | HMDB00157 | 16.4 | 265 | 280 | 73 | 1481 | *(21)* |
| Citric acid | Tricarboxylic acids and derivatives | HMDB00094 | 16.6 | 273 | 147 | 347 | 1494 | (21) |
| 1,5-Anhydroglucitol | CARBs and CARB conjugates | HMDB02712 | 16.9 | 217 | 191 | 129 | 1550 | *(21)* |
| Pyranose 1 ((allose 1/ mannose 1) | CARBs and CARB conjugates | HMDB00169 | 17.0 | 319 | 205 | 147 | 1580 | *(21)* |
| Pyranose 2 (glucose 1/altrose 1/ galactose 1/talose 1) | CARBs and CARB conjugates | HMDB00143 | 17.3 | 319 | 205 | 147 | 1594 | *(21)* |
| Pyranose 3 (talose 2 / glucose 2) | CARBs and CARB conjugates | HMDB00122 | 17.4 | 205 | 319 | 147 | 1580 | *(21)* |
| Pyranose 4 (altrose 2) | CARBs and CARB conjugates | HMDB01151 | 17.5 | 73 | 319 | 205 | 1586 | *(21)* |
| Histidine | AAs, peptides, and analogues | HMDB00177 | 17.5 | 154 | 73 | 254 | 1594 | *(21)* |
| Glucuronic acid | CARBs and CARB conjugates | HMDB00169/ HMDB00127 | 17.6 | 160 | 147 | 160 | 1603 | *(21)* |
| Tyrosine | AAs, peptides, and analogues | HMDB00158 | 17.8 | 220 | 280 | 280 | 1613 | *(21)* |
| Ascorbic acid | Furanones | HMDB00044 | 17.9 | 332 | 147 | 332 | 1621 | *(21)* |
| Palmitoleic acid | FAs and conjugates | HMDB12328 | 18.7 | 311 | 75 | 311 | 1702 | *(39)* |
| Palmitic acid | FAs and conjugates | HMDB00220 | 18.9 | 317 | 313 | 129 | 1720 | *(21)* |
| Myo-inositol | Alcohols and polyols | HMDB00211 | 19.3 | 305 | 318 | 191 | 1768 | *(21)* |
| Uric acid | Purines and purine derivatives | HMDB00289 | 19.4 | 441 | 73 | 456 | 1768 | (21) |
| Llinoleic acid | Lineolic acids and derivatives | HMDB00673 | 20.5 | 75 | 67 | 55 | 1885 | *(39)* |
| Tryptophan | Indolyl carboxylic acids and derivatives | HMDB00929 | 20.5 | 202 | 73 | 291 | 1889 | *(21)* |
| Elaidic acid | FAs and conjugates | HMDB00573 | 20.6 | 339 | 129 | 117 | 1886 | *(39)* |
| Oleic acid | FAs and conjugates | HMDB00207 | 20.6 | 339 | 117 | 339 | 1892 | *(39)* |
| Trans-13-octadecenoic acid | FAs and conjugates | HMDB41480 | 20.7 | 129 | 117 | 145 | 1895 | *(39)* |
| Stearic acid | FAs and conjugates | HMDB00827 | 20.8 | 341 | 75 | 129 | 1916 | *(39)* |
| Xanthotoxin | Furanocoumarins | HMDB14693 | 20.9 | 129 | 117 | 73 | 1916 | *(39)* |
| 5-hydroxy-L-tryptophan | Tryptamines and derivatives | HMDB00472 | 22.3 | 290 | 73 | - | 2114 | *(102)* |
| Cholesterol | Cholestane steroids | HMDB00067 | 27.8 | 329 | 73 | 368 | 2827 | *(21)* |
