# Supplementary material for: Optimization of a GC-MS method for the profiling of microbiota-dependent metabolites in blood samples: An application to type 2 diabetes and prediabetes
Source: Front Mol Biosci. 2022 Sep 23;9:982672. doi: 10.3389/fmolb.2022.982672 (PMC9538375; doi:10.3389/fmolb.2022.982672)
Supplement: Supplementary file 2 [file Table2.docx]

**Table S2.** Repeatability and reproducibility of MDM measurements.

| **Name** | **Repeatability  in plasma (n=50)** | **Repeatability  in serum  (n=50)** | **Reproducibility  in plasma  (n=10, s=3)** | **Reproducibility  in serum  (n=10, s=3)** |
| --- | --- | --- | --- | --- |
| Pyruvic acid | 5.0 | 8.7 | 15.1 | 16.2 |
| Lactic acid | 3.5 | 9.3 | 8.5 | 2.4 |
| Glycolic acid | 11.1 | 11.7 | 20.3 | 23.0 |
| Valine sum | 10.6 | 7.1 | 25.2 | 12.7 |
| Alanine | 17.1 | 10.0 | 15.0 | 17.5 |
| 3-methyl-2-oxobutanoic acid | 11.8 | 12.0 | >30 | >30 |
| Acetoacetate | 16.4 | 10.3 | >30 | >30 |
| Glycine | 17.8 | 9.0 | 18.5 | 15.9 |
| α-Hydroxybutyric acid | 5.4 | 5.2 | 18.9 | 14.9 |
| Oxalic acid | 9.6 | 13.1 | 20.0 | 22.8 |
| p-cresol | 8.5 | 10.6 | 23.3 | 21.1 |
| 3-Hydroxybutyric acid | 17.9 | 20.4 | 28.4 | >30 |
| Leucine | 8.4 | 5.4 | 24.8 | 12.7 |
| Isolucine | 8.9 | 21.1 | 25.4 | 12.4 |
| N-methylalanine | 13.3 | 22.9 | 21.6 | 16.2 |
| Proline | 20.5 | 8.3 | 26.6 | 9.2 |
| 2-ketoisocaproic acid | 7.5 | 7.0 | 13.6 | 9.0 |
| Urea | 4.5 | 5.6 | 29.1 | 25.1 |
| Benzoic Acid | >30 | >30 | >30 | >30 |
| Ethanolamine | 11.8 | 17.0 | 20.8 | 10.1 |
| Glycerol | 3.8 | 14.9 | 16.5 | >30 |
| Phosphoric acid | 5.6 | 17.3 | 24.5 | 20.2 |
| Succinic acid | 11.2 | 9.3 | 27.2 | 20.5 |
| Glyceric acid | 9.8 | 10.7 | 18.3 | 18.7 |
| Fumaric acid | 16.2 | 10.8 | 20.7 | >30 |
| Serine | 18.0 | 5.0 | 16.2 | 17.0 |
| Nonanoic acid | 11.0 | 16.2 | >30 | >30 |
| Threonine | 17.3 | 4.6 | 14.9 | 14.4 |
| 2-Aminomalonic acid | 20.4 | >30 | >30 | >30 |
| Aspartic acid | 28.7 | 21.9 | >30 | >30 |
| Malic acid | 10.9 | 9.7 | 11.1 | 12.4 |
| Threitol | 8.2 | 8.5 | 14.4 | 18.6 |
| Methionine | 17.6 | 9.5 | 18.9 | 20.6 |
| Glutamic acid | 10.7 | 8.0 | 11.8 | 15.9 |
| 5-Oxoproline | 10.7 | 8.1 | 11.8 | 15.9 |
| Trans-4-hydroxy-L-proline | 18.0 | 7.9 | >30 | 12.8 |
| Aspartic acid | 15.6 | >30 | 23.6 | >30 |
| Iminodiacetic acid | 15.6 | >30 | 23.4 | >30 |
| 2-Aminoadipic acid | >30 | 25.8 | 28.0 | >30 |
| Cysteine | 24.6 | 14.4 | >30 | 22.3 |
| Creatinine | 15.0 | 8.5 | >30 | 24.0 |
| Threonic acid | 10.7 | 9.5 | 12.5 | 27.6 |
| Ketoglutaric acid | 18.9 | 17.1 | 25.6 | >30 |
| Glutamic acid | 17.9 | 15.4 | 22.1 | 12.4 |
| Phenylalanine | 15.5 | 7.2 | 24.6 | 19.3 |
| Pyrophosphate | 19.0 | >30 | >30 | >30 |
| Lauric acid | 24.1 | 21.3 | >30 | 26.8 |
| Asparagine | 26.0 | 22.2 | >30 | 12.2 |
| Lysine | 25.9 | 18.1 | >30 | >30 |
| Glutamine | >30 | 25.8 | >30 | 22.6 |
| Ornithine | >30 | >30 | >30 | >30 |
| Hypoxanthine | 11.6 | 11.7 | 14.7 | >30 |
| Citric acid | 12.4 | 7.6 | 20.6 | >30 |
| 1,5-Anhydroglucitol | 3.7 | 4.7 | 17.1 | 28.2 |
| Pyranose 1 ((allose 1/ mannose 1) | 7.1 | 9.2 | 16.2 | >30 |
| Pyranose 2 (glucose 1/altrose 1/ galactose 1/talose 1) | 7.2 | 14.3 | 17.4 | 19.8 |
| Pyranose 3 (talose 2 / glucose 2) | 6.5 | 10.6 | 13.0 | >30 |
| Pyranose 4 (altrose 2) | 5.7 | 11.1 | 16.9 | 6.9 |
| Histidine | 11.2 | 9.3 | 17.0 | 18.0 |
| Glucuronic acid | 8.9 | 9.9 | 13.4 | >30 |
| Tyrosine | 15.0 | 8.7 | >30 | 7.9 |
| Ascorbic acid | 17.3 | >30 | 19.8 | 22.3 |
| Palmitelaidic acid | 11.7 | 12.9 | 21.4 | >30 |
| Palmitic acid | 7.8 | 7.2 | >30 | >30 |
| Myo-inositol | 7.9 | 6.4 | 14.2 | 13.0 |
| Uric acid | 19.1 | 19.4 | >30 | >30 |
| Linoleic acid | 19.6 | 20.2 | >30 | >30 |
| Tryptophan | 19.6 | 16.9 | >30 | 28.3 |
| Elaidic acid | 20.0 | 15.7 | >30 | >30 |
| Oleic acid | 12.9 | 15.8 | >30 | >30 |
| Trans-13-octadecenoic acid | 15.1 | 16.1 | >30 | >30 |
| Stearic acid | 10.0 | 8.4 | 28.0 | >30 |
| Xanthotoxin | 10.0 | 8.4 | 28.0 | >30 |
| 5-hydroxy-L-tryptophan | 7.2 | >30 | 8.6 | >30 |
| Cholesterol | 18.7 | 17.0 | >30 | 23.0 |
