# Supplementary material for: Optimization of a GC-MS method for the profiling of microbiota-dependent metabolites in blood samples: An application to type 2 diabetes and prediabetes
Source: Front Mol Biosci. 2022 Sep 23;9:982672. doi: 10.3389/fmolb.2022.982672 (PMC9538375; doi:10.3389/fmolb.2022.982672)
Supplement: Supplementary file 3 [file Table3.docx]

**Table S3.** Stability of MDMs in both matrices.

| **Metabolites** | **Stability MDMs for plasma (n=3)** | | | | **Stability MDMs for serum (n=3)** | | | |
| --- | --- | --- | --- | --- | --- | --- | --- | --- |
|  | **8 h** | **24 h** | **36 h** | **48 h** | **8 h** | **24 h** | **36 h** | **48 h** |
| Pyruvic acid | 0.6 | 3.8 | 5.2 | 7.5 | 3.6 | 2.6 | 3.1 | 5.7 |
| Lactic acid | 4.2 | 5.6 | 5.4 | 5.4 | 5.7 | 2.4 | 2.1 | 2.8 |
| Glycolic acid | 0.4 | 20.6 | 7.4 | 8.7 | 12.3 | 8.7 | 1.6 | 9.0 |
| Valine sum | 8.6 | 13.0 | 9.1 | 13.7 | 2.9 | 6.5 | 10.0 | 2.3 |
| Alanine | 15.5 | 19.0 | 10.2 | >30 | 1.9 | 1.8 | 3.1 | 3.9 |
| 3-methyl-2-oxobutanoic acid | 9.9 | 4.1 | 14.9 | 13.2 | 12.5 | 7.7 | 12.2 | 13.8 |
| Acetoacetate | 22.1 | 20.0 | 11.7 | 14.5 | 6.2 | 12.0 | 13.5 | >30 |
| Glycine | 15.0 | 12.3 | 12.0 | >30 | 1.3 | 4.3 | 4.0 | 7.6 |
| α-Hydroxybutyric acid | 3.6 | 8.4 | 5.5 | 5.7 | 4.0 | 5.1 | 6.3 | 2.1 |
| Oxalic acid | 16.5 | 9.7 | 15.5 | 5.6 | 1.8 | 2.1 | 7.9 | 4.5 |
| p-cresol | 8.4 | 10.5 | 3.6 | 22.2 | 3.4 | 8.6 | 22.0 | 12.6 |
| 3-Hydroxybutyric acid | 18.3 | 17.1 | 15.7 | 6.7 | 14.0 | 14.2 | 25.7 | >30 |
| Leucine | 7.5 | 11.1 | 7.4 | 7.6 | 4.0 | 6.4 | 9.9 | 3.1 |
| Isolucine | 9.5 | 9.6 | 6.3 | 12.8 | 3.4 | 5.3 | 10.0 | 3.7 |
| N-methylalanine | 9.5 | 9.6 | 6.3 | 12.8 | 14.6 | 2.4 | 10.4 | 1.2 |
| Proline | 11.1 | 15.6 | 10.0 | 17.3 | 3.8 | 4.9 | 4.3 | 2.5 |
| 2-ketoisocaproic acid sum | 11.1 | 15.6 | 10.0 | 17.3 | 4.6 | 0.3 | 12.4 | 7.6 |
| Urea | 16.9 | 23.1 | 12.2 | >30 | 1.3 | 3.5 | 6.5 | 3.3 |
| Benzoic Acid | 3.4 | 4.2 | 4.7 | 6.6 | >30 | >30 | >30 | >30 |
| Ethanolamine | 2.3 | 4.8 | 4.8 | 8.3 | 7.2 | 25.0 | 26.1 | 17.8 |
| Glycerol | 17.5 | 20.5 | 21.2 | >30 | 2.7 | 3.2 | 4.6 | 7.2 |
| Phosphoric acid | 3.3 | 5.2 | 9.9 | 10.8 | 10.1 | 3.4 | 2.1 | >30 |
| Succinic acid | 3.0 | 4.2 | 5.1 | 4.0 | 13.0 | 9.6 | 11.6 | 4.0 |
| Glyceric acid | 4.2 | 8.3 | 4.5 | 6.7 | 7.4 | 12.4 | 11.5 | 1.9 |
| Fumaric acid | 1.6 | 21.0 | 7.5 | 8.5 | 9.3 | 12.0 | 10.1 | 11.0 |
| Serine | 12.5 | 3.7 | 8.1 | 6.2 | 1.4 | 5.9 | 3.4 | 4.0 |
| Nonanoic acid | 20.8 | 25.7 | 19.4 | 6.2 | 5.9 | 10.2 | 10.7 | 20.0 |
| Threonine | 13.5 | 19.0 | 10.0 | >30 | 2.7 | 4.5 | 1.1 | 1.5 |
| 2-Aminomalonic acid | 6.9 | 4.2 | 4.9 | 19.5 | 17.2 | 28.4 | 23.5 | >30 |
| Aspartic acid | 13.4 | 19.6 | 9.3 | >30 | 27.0 | 20.1 | 10.6 | 10.3 |
| Malic acid | 26.3 | 25.7 | 12.1 | 5.3 | 18.7 | 10.1 | 8.2 | 11.1 |
| Threitol | 5.0 | 12.4 | 20.8 | >30 | 4.8 | 4.2 | 5.6 | 4.1 |
| Methionine | 6.6 | 11.8 | 9.7 | 8.5 | 2.3 | 3.3 | 3.7 | 2.4 |
| Glutamic acid | 3.0 | 13.5 | 3.7 | 13.6 | 8.5 | 9.8 | 4.9 | 11.9 |
| 5-Oxoproline | 9.6 | 15.9 | 9.0 | >30 | 8.5 | 9.8 | 4.9 | 11.9 |
| Trans-4-hydroxy-L-proline | 3.2 | 7.6 | 7.1 | 13.0 | 2.1 | 7.1 | 4.2 | 5.0 |
| Aspartic acid | 3.2 | 7.6 | 7.1 | 12.9 | 18.6 | 10.3 | >30 | >30 |
| Iminodiacetic acid | 8.2 | 24.3 | 12.2 | >30 | 18.6 | 10.3 | >30 | >30 |
| 2-Aminoadipic acid | 15.6 | 15.2 | 7.7 | >30 | 14.7 | 25.7 | >30 | >30 |
| Cysteine | 15.6 | 15.2 | 7.7 | >30 | 13.0 | 10.2 | 11.6 | 6.8 |
| Creatinine | 28.2 | 7.0 | 20.5 | 12.1 | 4.5 | 10.5 | 7.1 | 2.4 |
| Threonic acid | 13.3 | 28.2 | 12.8 | >30 | 8.9 | 12.9 | 12.9 | 13.4 |
| Ketoglutaric acid | 20.9 | 5.6 | 21.4 | 12.4 | 18.6 | 7.6 | 0.8 | 23.1 |
| Glutamic acid | 10.4 | 12.3 | 9.8 | 8.8 | 0.8 | 6.2 | 0.7 | >30 |
| Phenylalanine | 21.3 | 10.1 | 6.7 | 5.0 | 6.0 | 3.6 | 2.1 | 1.2 |
| Pyrophosphate | 11.8 | 21.9 | 8.4 | >30 | 26.5 | 22.5 | 25.3 | >30 |
| Lauric acid | 8.3 | 11.7 | 6.1 | >30 | 13.0 | 4.2 | 27.8 | 6.9 |
| Asparagine | 8.7 | 24.2 | 6.7 | 11.2 | 2.6 | 17.7 | 1.5 | >30 |
| Lysine | 11.1 | 8.8 | 15.3 | >30 | 12.3 | >30 | >30 | >30 |
| Glutamine | 11.0 | 10.5 | >30 | >30 | 5.9 | 9.3 | 8.1 | >30 |
| Ornithine | >30 | >30 | >30 | >30 | 7.4 | 13.2 | >30 | >30 |
| Hypoxanthine | 15.7 | 12.8 | >30 | >30 | 16.1 | 1.6 | 7.8 | 3.2 |
| Citric acid | 16.4 | 3.6 | >30 | >30 | 3.9 | 4.2 | 5.6 | 2.6 |
| 1,5-Anhydroglucitol | 3.7 | 7.7 | 11.1 | >30 | 3.6 | 4.5 | 7.0 | 1.5 |
| Pyranose 1 ((allose 1/ mannose 1) | 9.1 | 20.6 | 6.4 | 9.6 | 2.5 | 3.1 | 1.9 | 2.1 |
| Pyranose 2 (glucose 1/altrose 1/ galactose 1/talose 1) | 2.6 | 2.3 | 5.4 | 5.2 | 7.4 | 0.4 | 2.7 | >30 |
| Pyranose 3 (talose 2 / glucose 2) | 2.9 | 3.0 | 3.2 | 6.9 | 8.3 | 1.0 | 2.9 | 2.4 |
| Pyranose 4 (altrose 2) | 5.6 | 5.0 | 3.7 | 6.8 | 7.1 | 0.1 | 2.2 | 2.0 |
| Histidine | 5.7 | 3.0 | 6.4 | 3.5 | 4.5 | 6.6 | 2.1 | 5.8 |
| Glucuronic acid | 5.3 | 2.4 | 5.7 | 2.5 | 11.1 | 3.5 | 4.5 | 3.5 |
| Tyrosine | 3.3 | 10.4 | 0.9 | 14.5 | 5.4 | 6.0 | 3.2 | 4.6 |
| Ascorbic acid | 6.9 | 4.9 | 8.1 | 6.2 | 12.0 | 11.0 | 13.3 | 3.1 |
| Palmitelaidic acid | 6.8 | 15.2 | 6.6 | 53.3 | 10.2 | 15.0 | 15.6 | 11.8 |
| Palmitic acid | 4.5 | 27.4 | 46.6 | 51.7 | 1.5 | 3.5 | 8.9 | 1.5 |
| Myo-inositol | 6.4 | 16.1 | 0.7 | 26.3 | 9.9 | 3.0 | 9.7 | 3.8 |
| Uric acid | 11.0 | 9.6 | 15.0 | 8.0 | 12.7 | 3.0 | 20.7 | 8.9 |
| Linoleic acid | 4.6 | 2.3 | 5.0 | 2.6 | 11.4 | 6.6 | 15.7 | >30 |
| Tryptophan | 11.4 | 15.8 | 8.6 | 10.4 | 8.3 | 5.9 | 9.1 | 5.2 |
| Elaidic acid | 14.3 | 24.0 | 13.3 | 87.3 | 11.7 | 13.3 | 19.6 | 16.3 |
| Oleic acid | 23.7 | 10.1 | 27.6 | 52.6 | 11.7 | 13.3 | 19.6 | 16.3 |
| Trans-13-octadecenoic acid | 8.1 | 21.0 | 5.7 | 48.7 | 11.7 | 13.3 | 20.9 | 16.4 |
| Stearic acid | 12.5 | 11.0 | 16.4 | 4.8 | 5.1 | 1.9 | 10.0 | 4.1 |
| Xanthotoxin | 12.5 | 11.0 | 16.4 | 4.7 | 5.1 | 1.9 | 10.0 | 4.1 |
| 5-hydroxy-L-tryptophan | 12.5 | 11.0 | 16.4 | 4.7 | 26.2 | >30 | >30 | >30 |
| Cholesterol | 10.3 | 8.4 | 6.0 | 10.1 | 26.4 | 21.3 | 13.3 | 17.2 |
